# Modeling *Edar* expression reveals the hidden dynamics of tooth signaling center patterning

**DOI:** 10.1371/journal.pbio.3000064

**Published:** 2019-02-07

**Authors:** Alexa Sadier, Monika Twarogowska, Klara Steklikova, Luke Hayden, Anne Lambert, Pascal Schneider, Vincent Laudet, Maria Hovorakova, Vincent Calvez, Sophie Pantalacci

**Affiliations:** 1 Laboratoire de Biologie et Modélisation de la Cellule, Université de Lyon, ENS de Lyon, Univ Claude Bernard, CNRS UMR 5239, INSERM U1210, Lyon, France; 2 Institut de Génomique Fonctionnelle de Lyon, Université de Lyon, ENS de Lyon, Univ Claude Bernard, CNRS UMR 5239, Lyon, France; 3 Unité de Mathématiques Pures et Appliquées, project team Inria NUMED, Université de Lyon, ENS de Lyon, CNRS UMR 5669, Lyon, France; 4 Institute of Experimental Medicine, The Czech Academy of Sciences, Prague, Czech Republic; 5 Department of Cell Biology, Faculty of Science, Charles University, Prague, Czech Republic; 6 Department of Biochemistry, University of Lausanne, CH-1066 Epalinges, Switzerland; 7 Institut Camille Jordan, Université de Lyon, Université Claude Bernard, CNRS UMR 5208, Lyon, France; Cancer Research UK London Research Laboratories, UNITED KINGDOM

## Abstract

When patterns are set during embryogenesis, it is expected that they are straightly established rather than subsequently modified. The patterning of the three mouse molars is, however, far from straight, likely as a result of mouse evolutionary history. The first-formed tooth signaling centers, called MS and R2, disappear before driving tooth formation and are thought to be vestiges of the premolars found in mouse ancestors. Moreover, the mature signaling center of the first molar (M1) is formed from the fusion of two signaling centers (R2 and early M1). Here, we report that broad activation of *Edar* expression precedes its spatial restriction to tooth signaling centers. This reveals a hidden two-step patterning process for tooth signaling centers, which was modeled with a single activator–inhibitor pair subject to reaction–diffusion (RD). The study of *Edar* expression also unveiled successive phases of signaling center formation, erasing, recovering, and fusion. Our model, in which R2 signaling center is not intrinsically defective but erased by the broad activation preceding M1 signaling center formation, predicted the surprising rescue of R2 in *Edar* mutant mice, where activation is reduced. The importance of this R2–M1 interaction was confirmed by ex vivo cultures showing that R2 is capable of forming a tooth. Finally, by introducing chemotaxis as a secondary process to RD, we recapitulated in silico different conditions in which R2 and M1 centers fuse or not. In conclusion, pattern formation in the mouse molar field relies on basic mechanisms whose dynamics produce embryonic patterns that are plastic objects rather than fixed end points.

## Abstract 2

The generation of patterns during development is generally viewed as a direct process, but this study of mouse tooth development shows how embryonic patterns can be constructed and erased in a very dynamic manner before the correct pattern is achieved.

## Author summary

Organs, such as teeth, that form regular patterns are of particular interest to developmental biologists. These patterns are established early in the embryo, and it has generally been thought the organs appear in what is their final position. Recent studies that focus on the dynamics of patterning events challenge this view, suggesting that pattern formation can be more complex than previously thought. For example, mouse molars form from “organizing centers,” which appear, disappear, or fuse in a complex sequence of events, until the final pattern is stabilized. Based on the dynamics of expression of the *Edar* gene, we built a mathematical model of how tooth “organizing centers” form. We reveal that a newly formed organizing center can actively impair or erase a previously formed one. We call this phenomenon a developmental “palimpsest,” from the terminology of old manuscripts that were scraped to be reused again. This indirect developmental process likely reflects the evolutionary history of mice, which lost premolars while maintaining their embryonic organizing centers. More broadly, we believe that overwriting or correcting previously established patterns during development might be more common than anticipated, simply due to the fact that developmental programs are modified by incrementation during evolution.

## Introduction

The emergence of ordered patterns in multicellular organisms has been a major field of research in developmental biology, revealing a diversity of pattern formation mechanisms. While some patterns appear simultaneously (e.g., *Drosophila* segments, mouse hair), others appear sequentially (e.g., feathers on chicken’s back), most often as the structure grows distally (e.g., short-germ insects’ segments, somites, limbs proximodistal elements, palatal rugae). Several types of patterning mechanisms have been proposed. Some rely on a prepattern, like the “positional information,” model in which a gradient of a signaling molecule is turned into a more complex pattern by interpreting the varying concentration at each position in space [1,2]. Others rely on self-organization, resulting in spontaneous pattern formation as seen in reaction–diffusion (RD) (Turing) mechanisms or upon chemotaxis (see below and [3–5]). Depending on the mechanism, temporal dynamics of pattern formation have been more or less emphasized. Sequential formation requires the consideration of temporal aspects that can be neglected when the pattern forms at a glance [6,7]. Spontaneous pattern formation results from the internal dynamics of the system, which naturally places the focus on the temporal dynamics. For example, the work of Salazar-Ciudad and Jernvall has emphasized the role for temporal changes in system conditions during 3D morphogenesis when patterning and growth are coupled: patterning at time *t* modifies the 3D geometry of the system through growth, and this will influence downstream patterning at time *t* + 1 [7,8]. In contrast, positional information has been mostly associated with static representations, for example, in the French flag model [2,3]. In most cases, however, patterning is viewed as a directional temporal process: from a prepattern or a spatial heterogeneity emerges the final pattern, which is then stabilized. It is, however, questionable whether biological systems, which result from a historical, contingent process, proceed in such a directional manner, or if transient patterns can be constructed and deconstructed during embryogenesis until the final pattern is formed. Recently, a careful reexamination of the example of simultaneous pattern formation, namely the formation of *Drosophila* gap gene expression pattern, revealed that, as maternal inputs decay, gene expression patterns change with important consequences for the final pattern [9]. To our knowledge, other examples are lacking. Here, we studied the question in the model of sequential patterning of mouse molars.

The search for the general mechanisms generating patterns in biology has been greatly influenced by the theoretical work of the mathematician Alan Turing [4,5,10]. The generalization of this work has led to many classes of RD mechanisms, in which two (or more) molecules characterized by a different spatial range of action and a given topology of interaction can self-organize a stable pattern but also exhibit behaviors such as oscillations or propagating waves [4]. The most iconic example is the case in which a short-range activator that self-amplifies and activates its own long-range inhibitor can create spots, stripes, or labyrinths. Recently, it has been shown that many biological systems such as hair and feathers [11,12], the rugae of the palate [13], digits [14], and somites [15] exhibit features of RD mechanisms. This should not be taken too strictly, however. Geirer and Meinhardt pointed out that any process involving local self-enhancement and lateral inhibition has the potential to drive spontaneous pattern formation [16]. For example, color pattern formation in zebrafish can be explained by RD models but at least partly involves cell interactions rather than the diffusion of biomolecules [17,18]. Pattern formation can also arise from purely chemotaxis-mediated self-organization. When cell movement is driven by concentration gradients of chemotactic cues, positive feedbacks between cell density and chemo-attractant production are known to enhance the local concentration of cells and may result in self-sustained aggregation [19–21]. Chemotaxis plays a prominent role in feather formation [22], and this is likely also the case in most other epithelial appendages such as hair [11].

Mouse molars are a good example of repeated structures that form through sequential pattern formation. Mice have only three molars per quadrant, separated from incisors by a diastema, as canines and premolars have been lost in the evolution of mouse lineage [23]. Molars develop sequentially from the first, most anterior molar (the first molar, M1) to the third, most posterior (the third molar, M3). They develop from a cylinder-shaped invagination of the dental lamina, the so-called dental epithelium [24–26], where tooth-specific signaling centers, called primary enamel knots (PEKs), are patterned. PEK formation in the epithelium requires signaling from both the epithelium and the mesenchyme [27], including a mechanical signal induced by mesenchyme condensation [28]. These signaling centers then drive the formation of individual teeth by promoting “cap” formation, the process by which the underlying condensed mesenchyme gets surrounded by the epithelium. Indirect evidence that activation-inhibition mechanisms determine sequential formation of these signaling centers comes from the similarity of tooth formation to other epithelial appendages [29], namely hair and palatal rugae, whose patterning is clearly ruled by Turing-type mechanisms [11,13]. The most direct evidence is a study by Kavanagh and colleagues [30], showing that when tissue that will form the second molar (M2) is separated from M1, M2 forms earlier and becomes larger. The earlier development of M2 is also seen when the developing molar row is stimulated with Bmp4 or ActivinβA, which are activators of tooth formation [30], or upon interference with Shh, an inhibitor of tooth formation [31]. This provides evidence that the first molar somehow acts as a source of inhibitor for the next forming molar. It is noteworthy that three genes encoding inhibitors of tooth development (Shh, Sostdc1, Follistatin) are found in three large genomic regions (quantitative trait loci) that affect the size relationships between molars [32].

**Fig 1:**
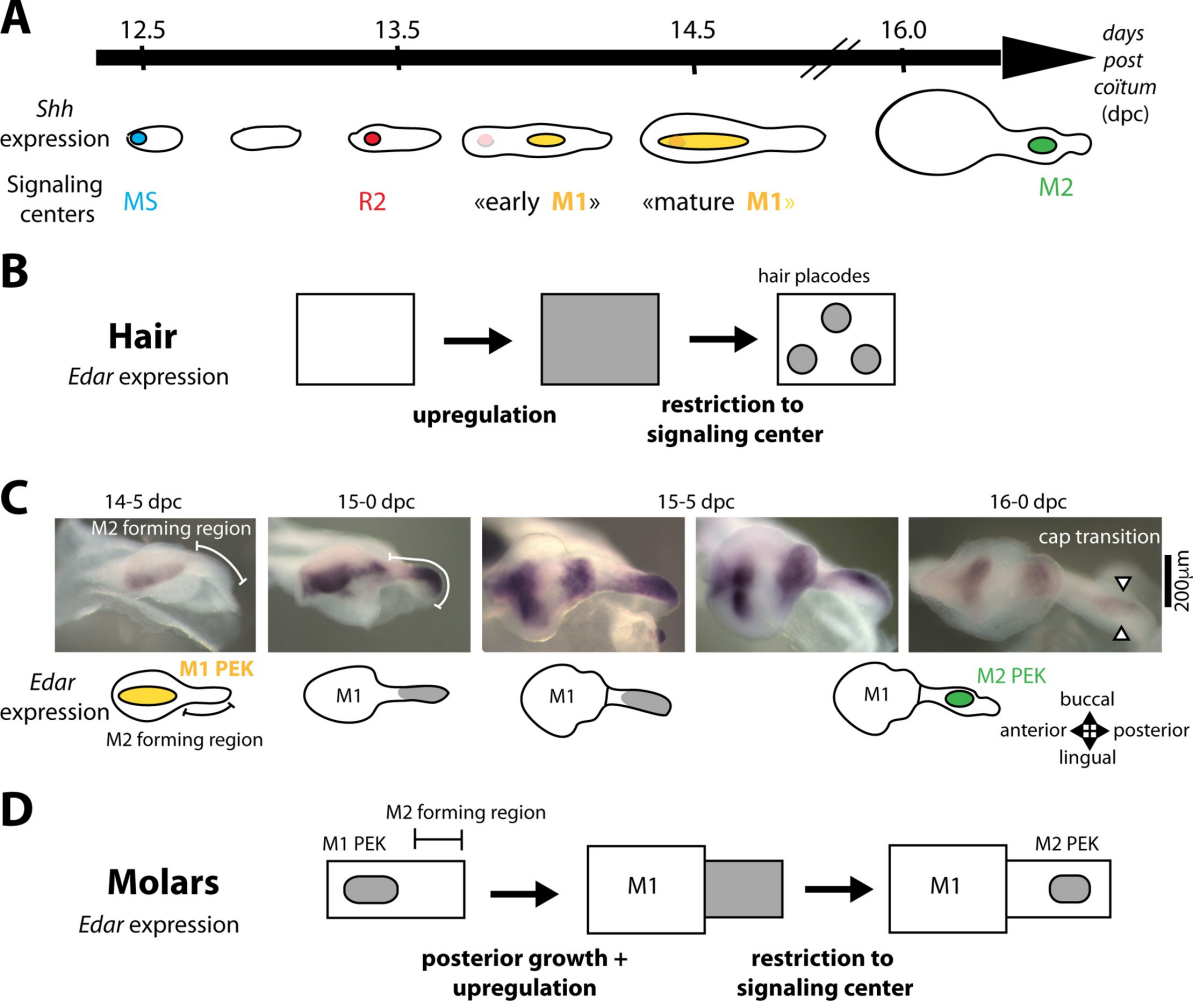
*Edar* expression is dynamically regulated during hair and tooth patterning. **(A)** Scheme summarizing the sequential patterning of signaling centers in the posteriorly growing dental epithelium, as revealed with *Shh* expression. At 12.5 dpc, a spot of *Shh* expression marks the rudimentary MS signaling center. *Shh* expression then disappears and around 13.5 dpc, a new spot of *Shh* expression marks the more posterior R2 signaling center. *Shh* expression decreases again but is occasionally transiently seen in R2, as the more posterior early M1 signaling center forms at 14 dpc. By 14.5 dpc, the mature M1 signaling center marked by *Shh* expression encompasses the former R2 and early M1 signaling centers. At 16.0 dpc, *Shh* marks the M2 signaling center. (B) Scheme showing *Edar* regulation in the epidermis during primary hair patterning. *Edar* becomes up-regulated in the forming hair placode while being down-regulated in its neighborhood, highlighting the outcome of the activation-inhibition mechanisms that patterned the placode and avoid further formation of placodes in the vicinity. (C) In situ hybridization showing *Edar* expression in the isolated dental epithelium from 14.5 to 16.0 days, a period corresponding to growth of the tail region (“M2 forming region”) and patterning of the M2 signaling center in this newly grown region (at 16.0). At 14.5, *Edar* is restricted to the PEK of the first molar (yellow on the scheme). At 15.0, *Edar* expression is seen in the posteriormost part of the tail, the M2-forming region (gray on the scheme). Between 15.5 and 16.0, it is restricted to the PEK of the second molar (M2, green on the scheme). On the scheme, sustained expression of *Edar* in the cusps of the maturating first molar has been omitted for simplicity. Pictures presented were selected from *n* > 100 samples harvested to cover the time period 14.5–16.0 dpc and time ordered according to embryonic body weight. All samples fit with the temporal evolution of *Edar* expression shown here, including intermediate stages. (D) Scheme summarizing the expression pattern in (C), to be compared with (B). The situation in hair is similar to the situation in the posterior part of the dental epithelium. dpc, days post coitum; MS, mesial segment; M1, first molar; M2, second molar; PEK, primary enamel knot; R2, rudiment 2.

The sequential patterning of mouse molars in the lower jaw (Fig 1A) involves two transient signaling centers [33] that fail to drive proper cap transition, yet form morphologically distinguishable buds (called MS for mesial segment, and R2 for rudiment 2) [33,34]. These buds might be vestiges of lost premolars [33,35]. Monitoring these signaling centers via *Shh* expression revealed that the first of these transient signaling centers called MS initiates sequential patterning and then disappears [33]. Subsequently, the R2 signaling center forms. As it starts vanishing, M1 early signaling forms posteriorly [33]. Soon after, R2 and M1–early signaling centers are encompassed in a giant *Shh*-expressing signaling center [33,36]. Here, we will refer to this large center as the mature M1 signaling center. A similar situation with two abortive buds (called R1 and R2) has been noticed in the upper jaw [26]. Their signaling centers have not yet been characterized, although they are morphologically more apparent than in the lower jaw.

Interestingly, mutations in genes affecting various developmental pathways (FGF, Shh, Wnt, BMP, and Eda pathways) lead to a supernumerary tooth in front of M1, as if it were a premolar [37]. Studies that specifically addressed the question found that the R2 signaling center was rescued and enabled to form a tooth [36,38–42]. The picture is thus fairly complex, especially because we lack direct evidence for the dynamics of activation-inhibition mechanisms that pattern signaling centers in the dental epithelium and promote tooth formation.

The Eda pathway has the potential to shed light on these mechanisms. It has a consistent role in activation-inhibition mechanisms in epithelial appendages as distinct as hair, feathers, and teeth [43,44]. This role was best studied during pattern formation of mouse guard hairs. The receptor of the pathway, Edar, is first broadly expressed in the epidermis. Concomitantly with patterning of hair signaling centers, it becomes up-regulated in the placodal signaling center and down-regulated in the neighborhood (Fig 1B). Without Edar signaling, no signaling center forms [12,45,46], while excessive signaling increases placode numbers and their packing density [12]. Current models posit that the Eda pathway is activated by Wnt, ActivinβA, and BMP4 pathways [12,46,47] but also feeds back on these and other pathways through the transcriptional activation of their diffusing ligands and inhibitors, for example, WNT10a/b, DKK4, CCN2/CTGF, Follistatin, and FGF20 [43,44]. More recently, Eda signaling was also shown to promote placodal fate by stimulating the centripetal aggregation of epithelial cells [48,49]. In teeth, the Eda pathway is dispensable for primary signaling center (PEK) formation, but is required for its correct sizing [49–51]. Similarly, it is necessary for correct patterning of the secondary signaling centers controlling cusp morphogenesis [41,50,52]. Although *Eda* and *Edar* mutants have reduced tooth size and cusp numbers, they sometimes form a small supernumerary tooth [53–55]. In gain-of-function mutations, an anterior supernumerary tooth is also found, and teeth are larger, with more cusps [52–54].

In this paper, we aimed at clarifying the temporal dynamics of signaling center formation in the dental epithelium. We studied the temporal dynamics of *Edar* gene expression, the receptor of the Eda pathway, during molar pattern formation and showed that it recalls the dynamics observed during hair patterning. Based on these data, we built an RD-type model of molar patterning that enables sequential signaling center formation and helps reveal the exquisitely complex temporal interactions leading to the construction and deconstruction of patterns in the developing molar row. Our model explains a counterintuitive result, the rescue of the abortive R2 bud in the inhibitory context of *Edar* loss of function. Finally, we show that Edar is necessary for the formation of a fused R2–M1 signaling center in the lower jaw only, possibly through a chemotactic effect. We thus showed that patterning is not direct, although it follows simple mathematical rules.

## Results

### *Edar* regulation highlights activation in the growing dental epithelium

To get insights into molar row patterning, we examined the regulation of the *Edar* gene (Fig 1B). Because the early period of molar row patterning is complicated by the presence of vestigial signaling centers, we first focused on the patterning of the second molar. Because *Edar* is exclusively expressed in the epithelium, we performed in situ hybridization on mandibular epithelium that had been dissociated from the mesenchyme, thus providing a 3D view of *Edar* expression. At 14.5 days post coitum (dpc), *Edar* expression is restricted to the primary signaling center (PEK) of the first molar, and no expression is seen in the second molar field, which looks like a "tail" (Fig 1C). At 15.0 dpc, the "tail" has elongated and *Edar* expression is up-regulated in its most posterior part. By late 15.5 dpc, it starts restricting to the M2 PEK, just before M2 cap transition occurs (at 16.0 dpc). The restriction was concomitant with *Shh* expression, starting in the PEK of M2 [33].

This dynamic of an initial broad up-regulation of *Edar* followed by its restriction to a signaling center is reminiscent of what happens during hair patterning (compare Fig 1B and 1D). It suggests that the decision to form a tooth signaling center in the growing molar field proceeds in two phases. First, the whole dental epithelium is activated. This activation results in broad *Edar* expression, which is so far the only gene whose expression pattern marks the epithelium competent to form a tooth. Second, activation gets restricted spatially and gives rise to a signaling center. This results in the focused expression of *Edar* and of many other genes, including *Shh*, which together are known as PEK genes. In this view, *Edar* expression is a readout of activation levels in the molar field: where activation is high enough, *Edar* is expressed. To further formalize these ideas, we built a mathematical model of activation in the dental epithelium that could recapitulate the expression pattern of *Edar*.

### Activation dynamics can be modeled by a transition from a bistable system to a Turing system in a growing domain

**Fig 2:**
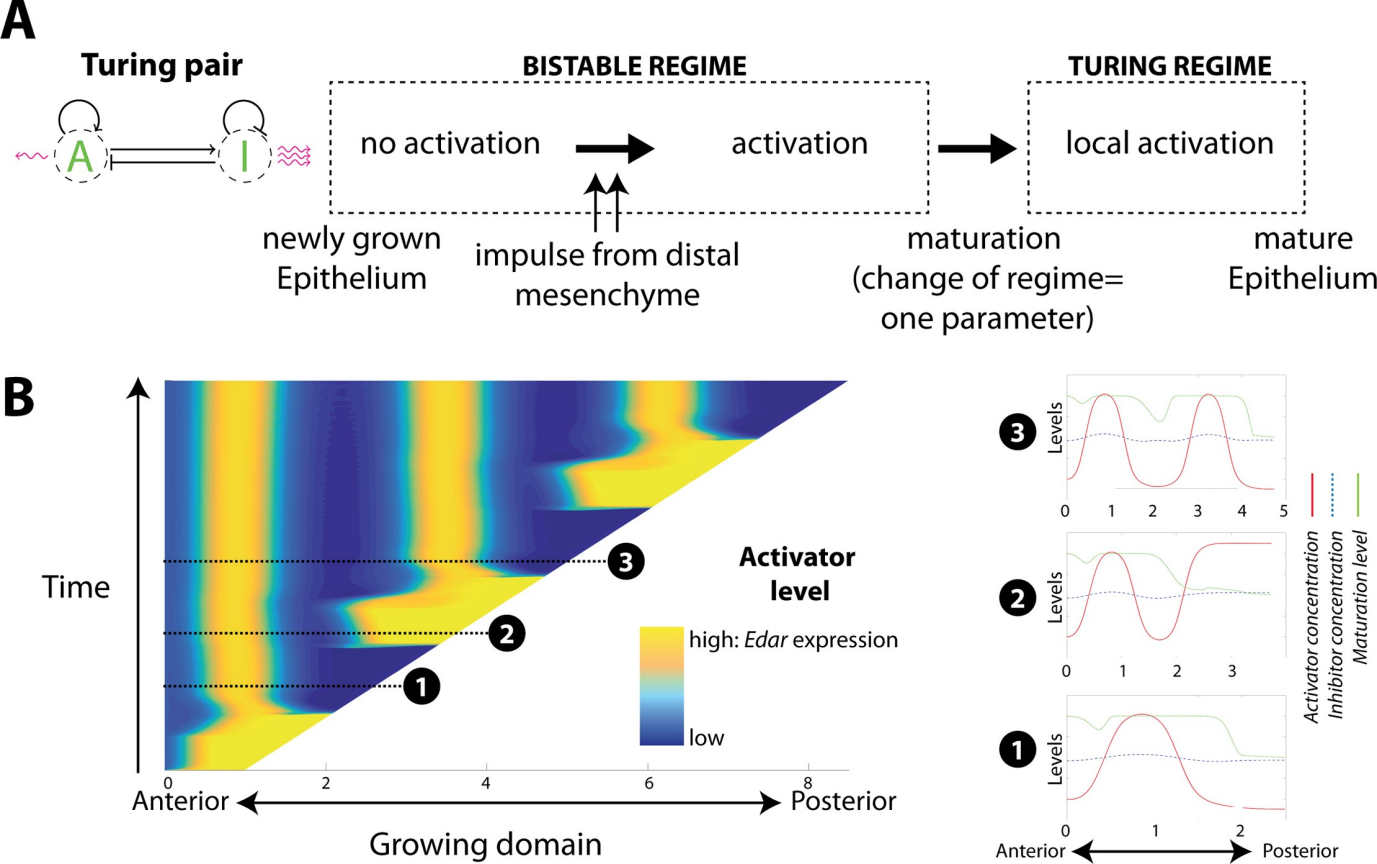
A model for signaling center patterning based on maturation-dependent shift from a bistable to a Turing regime. (A) Basic principles for a mathematical model of activation in the dental field, with *Edar* expression considered as a readout of activator concentration. The dental epithelium first transits from no activation to generalized activation, as the tissue is primed by the posterior mesenchyme. Activation induces tissue maturation, moving the system to a Turing regime, a switch that needs a single parameter change. Activation is then localized to the signaling center. (B) Modeling of the activation-inhibition mechanisms along the one-dimensional anteroposterior axis. Activator concentration is shown through space (*x*) and time (*y*) as the domain grows. Snapshots taken at three different time points are shown (red, activator concentration; blue, inhibitor concentration; green, domain maturation). The simulation shows the periodic behavior of the model: the dental epithelium grows in an inactivated state due to inhibition coming from the Turing spot (snapshot 1, see also Fig 1C, 14.5 dpc). Newly grown parts of the dental epithelium get activated on a time periodic basis (bright yellow posterior domains, snapshot 2, Fig 1C 15.0–15.5 dpc) and upon maturation, produce a Turing peak (snapshot 3, Fig 1C 16.0 dpc). The movie corresponding to the snapshots is available as S1 Movie. The model is described in detail in S1 Text. The code and this simulation can be found online (see Material and methods). Here, the regulation function in the activator-inhibitor system is symmetric, meaning that the low- and high-activation states are equally stable. dpc, days post coitum.

Switches between different activation states can be identified by *Edar* expression and suggest that Turing mechanisms occur in the dental epithelium: states first switch from no activation (no *Edar* expression) to broad activation (broad *Edar* expression) and then from broad activation to spatially restricted activation (focused *Edar* expression). From a mathematical point of view, these spatial behaviors can be modeled as two regimes of the same RD system (Fig 2A). The first regime, referred to in this paper as “the bistable regime,” admits two homogenous, stable states of a high and a low activation, respectively. This corresponds to the posterior part of the tissue, with transient up-regulation of *Edar*. Second, the so-called Turing regime is characterized by stable heterogeneous periodic patterns, which emerge from homogeneous patterns. In a minimal pair of reaction equations, the Turing regime is structurally close to the bistable regime (see Fig 1 in S1 Text). Switching between both regimes can be achieved by changing a single parameter value. Because the restricted expression is only seen in the developmentally advanced anterior part and is preceded by broad activation, we impose that the activator has a positive feedback on tissue maturation, resulting in the switch to the Turing regime. Biologically, this means that upon the broad wave of activation, new gene products have been released that modify the activation-inhibition parameters.

Our model follows the one-dimensional anteroposterior axis, with posterior domain growth. It describes the time evolution of concentration of an activator and its inhibitor, which diffuse with different speeds and undergo kinetic reactions (Fig 2A). A detailed description of the model and the parameters used in all simulations are found in the supplementary material (S1 Text). Below, we summarize the main characteristics of the model, which exhibits periodic behavior, as shown in a representative simulation (Fig 2, see also S1 Movie).

The tissue grows at its posterior end, and the newly produced tissue then matures exponentially in time, albeit at a slow rate. Maturation is stimulated in the presence of the activator, with some time delay. In zones where maturation reaches a threshold value, the system irreversibly switches from a bistable to a Turing regime. We postulate that this occurs because tissue maturation has an impact on the regulatory feedbacks of the activator-inhibitor system. We opted for a parsimonious modification: a decrease in the rate of auto-inhibition of the inhibitor in the mature tissue. This change of a single parameter value is sufficient to make the transition (Fig 2B, snapshots 2 to 3).

Before this switch can occur, such a simple system first needs to reach high levels of broad activation in the newly grown part (Fig 2B, snapshot 2). Based on the literature, and as further developed in the Discussion section, epithelia-mesenchyme interactions may play an important part in this broad activation, but through a mechanism that is largely unknown. Here, we simply assumed the existence of an extrinsic component representing the interaction with the mesenchyme. Below a certain threshold of activation, it will act to increase the concentration of the activator. Above a certain threshold, it will feed back negatively on it. This introduces an oscillatory behavior at the posterior end of the domain. Interestingly, although we do not explicitly put this in the model, the oscillatory behavior of the mesenchyme is spatially correlated to the growth of the domain. The transition from “no activation” to “activation” is promoted by the positive feedback from the mesenchyme but will only happen when the domain has grown enough to escape from the influence of the inhibitor from the Turing peak (Fig 2B, snapshot 1 to snapshot 2).

In summary, our theoretical model based on *Edar* expression involves activation-inhibition mechanisms in the dental epithelium, coupled with periodic activation of the growing dental epithelium.

### A developmental palimpsest occurs for MS and R2 patterning and can be modeled with a regime of traveling wave

**Fig 3:**
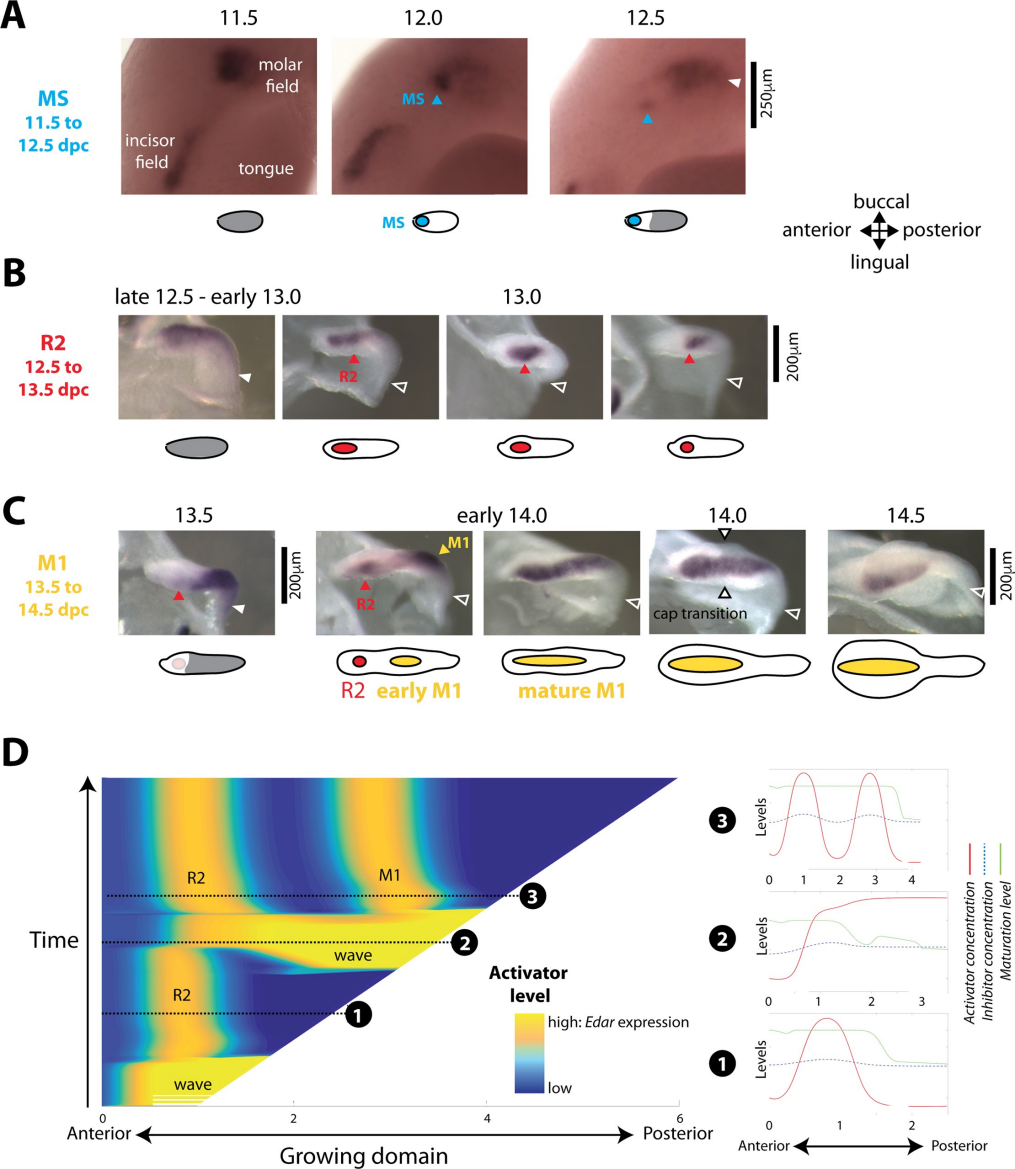
Pattern erasing (“developmental palimpsest”) in the early dental epithelium can be modeled with a traveling wave. **(**A, B, C) In situ hybridization with an *Edar* probe of whole mandibles (only one half is shown, [A]) or isolated dental epithelia (B, C) from 11.5 to 14.5 dpc. Pictures are shown with an accompanying scheme in which *Edar* broad expression is shown in gray and *Edar* focused expression is shown in color. Several rounds of *Edar* regulation are observed, culminating in the formation of a single signaling center. The first pattern of the MS signaling center (A) is erased by a new wave of *Edar* expression at late 12.5–13.0 dpc, resulting in the second pattern of the R2 signaling center (B, D), which in turn is erased by another wave of *Edar* expression at 13.5 dpc. A pattern with two spots is transiently observed that finally fuses in a single large spot corresponding to the mature M1 signaling center (C, D). Pictures presented were selected from *n* > 400 samples harvested to cover the time period 11.5–14.5 dpc and time ordered according to embryonic body weight. All samples fit with the temporal evolution of *Edar* expression shown here, including intermediate stages. (D) Pattern erasure can be observed in a modified model, in which we introduce an asymmetry in the bistable regime. The left panel shows the chronogram of a representative simulation (concentration of activator as a function of space (*x*) and time (*y*). Snapshots taken at three different time points are shown (red, activator concentration; blue, inhibitor concentration; green, domain maturation). Snapshot 1 corresponds to the R2 peak (13.0 dpc in panel B). Snapshot 2 shows the activation wave invading the R2 domain (13.5 dpc in panel C). Snapshot 3 shows recovery of the R2 peak, together with the newly formed M1 peak (early 14.0 dpc in panel C). The movie corresponding to the snapshots is available as S2 Movie. The model is described in detail in S1 Text. The code and this simulation can be found on MT github (see Material and methods). Contrary to Fig 2B, we have introduced some asymmetry in the self-regulation of the activator favoring the high-activation state (parameters Q_h > Q_l), which triggers a propagating wave. This wave is able to invade the immature zone and to destabilize the pre-established Turing pattern. dpc, days post coitum; M1, first molar.

Next, we focused on the dynamics of *Edar* expression during the complex chain of patterning events (schematized in 1A) that precedes the formation of the M1 signaling center, also known as the PEK (yellow in Fig 1A and 1C). The dynamic was partly similar to that observed for M2 patterning, although MS and R2 signaling centers fail to drive cap formation and to form a tooth ([33] and Fig 1A). Indeed, broad *Edar* expression restricts to these signaling centers in late 12.5 embryos for MS and early 13.5 embryos for R2 (corresponding to Shh-signaling center at 12.7 and 13.3 dpc in [33] and Fig 1A). Following the restriction, *Edar* expression starts again from the posterior part of the dental epithelium (white arrowhead). However, in contrast to what was seen for M2 (Fig 1C, 15.0 dpc), the wave of *Edar* expression did not stop at a distance from the preceding signaling center. Rather, it invaded the whole dental epithelium, including its anteriormost part, thus erasing the previous Turing pattern made by MS and R2 signaling centers (Fig 3A and 3B, 12.5 dpc, and Fig 3C, 13.5 dpc). In the case of MS, a new signaling center is formed following the wave. Prochazka and colleagues [33] showed by DiI tracing that R2 is just slightly posterior to MS. In the case of R2, two signaling centers are seen following the wave: R2 recovers and the early M1 signaling center is newly formed. We call this phenomenon a developmental palimpsest, because a palimpsest is a manuscript page that has been scraped or washed off to be used again for a novel text. Here, a first Turing pattern, assimilated as a first text, is erased by the *Edar* expression wave that “scrapes the first text,” and a new pattern is formed, representing the novel text.

This behavior can be naturally achieved with our model, because waves are generic features of bistable RD systems. Indeed, they exhibit three genuine types of solutions: two constant homogeneous solutions in each of the two states (high activation or low activation), as well as spatially heterogeneous solutions connecting the two stable states and traveling in time. The occurrence of the traveling wave relies on the asymmetry between the two stable states. Furthermore, the direction of the wave is determined by the relative stability of the two states: from the most to the least stable. A representative simulation of this modified model is shown in Fig 3D (see also S2 Movie). The active state is more stable, so that once activation is primed in the posterior part, a wave activates progressively the inactive area (Fig 3D, snapshot 2). It is important to notice that, before wave initiation, the immature area is maintained naturally in the stable inactive state under the influence of the Turing initial peak. The wave can, however, propagate into the mature tissue and erase the previously formed Turing pattern (Fig 3D, snapshots 1 and 2). Then, as a consequence of tissue maturation subsequent to wave activation, two activation peaks are formed by a secondary Turing patterning (Fig 3D, snapshot 3). This would correspond to recovered R2 and to the newly formed M1 signaling center. For this palimpsest to occur, the wave that initiates in the immature (bistable) domain should interact with the stable pattern in the mature Turing domain. Both the activation wave and the Turing peak feature stability. As such, understanding their interaction is far from trivial. Several conditions must be fulfilled in order to observe a palimpsest in the numerical tests, which are reviewed in the Supporting information (S1 Text–section S3). In particular, we found that auto-inhibition, if increased in the bistable regime, strengthened the wave and favored the palimpsest. We also found that it is sensitive to the temporal dynamics. It requires a suitable synchronization between domain growth, posterior activation, wave speed, and maturation rate.

### Increasing inhibition in the model can explain the counterintuitive rescue of R2 signaling center in the *Edar*^*dlJ*^ mutant

Most of the numerous mutants in which a premolar-like tooth forms, supposedly from R2, have larger- or simply normally sized molar teeth. The teeth of loss-of-function mutants for the Eda pathway are poorly grown, yet a premolar-like tooth can form. As a rescue of R2 is counterintuitive in this context, we decided to re-examine one of these mutants (*Edar*^*downlessJ*^ [*Edar*^*dlJ*^]) in the light of *Edar* dynamics and of the present model.

**Fig 4:**
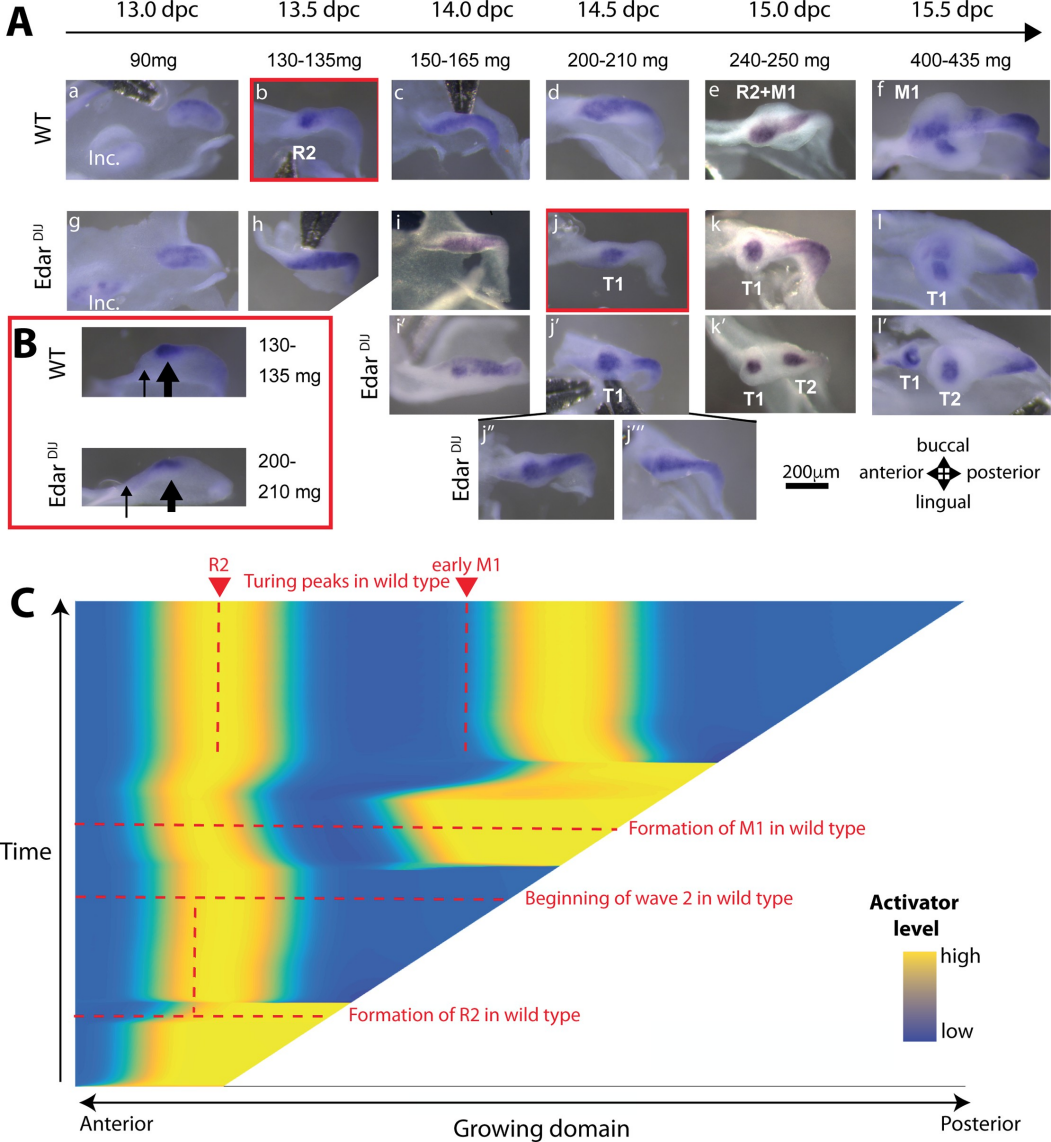
The changes in signaling center patterning observed in the *Edar*^*dlJ*^ mutant are predicted by the model. (A) In situ hybridization with an *Edar* probe in dissociated lower jaw epithelia from wild-type control (top row) and *Edar*^*dlJ/dlJ*^ mutant (second, third, and fourth rows) embryos of similar weight classes between 13.0 and 15.5 dpc. Patterning of the first signaling center (MS) occurs normally (see S1 Fig), but patterning of the second signaling center, called R2 in the wild type and T1 in the mutant, is delayed with variability (see also main text and S1 Table). This T1 signaling center is not erased and persists. (B) Side view of pictures outlined in red in panel A, showing that the T1 signaling center is found more posteriorly in the invaginated dental epithelium of the mutant, as compared with R2 in the wild type (thick arrows). Thin arrows point at the beginning of the dental epithelium invagination. (C) Same simulation as in Fig 3, but with increased inhibition. The position and timing of Turing peak formation in wild type (as in Fig 3E) are indicated with red dashed lines. Increasing the inhibition is sufficient to abolish the palimpsest and stabilize the first and second peaks. The model is described in detail in S1 Text. The code and this simulation can be found on MT github (see Material and methods). We used the same model and parameters as in Fig 3D, except for the inhibitor, whose rate of auto-inhibition was lowered to account for increased inhibition in the mutant. dpc, days post coitum; *Edar*^*dlJ*^, *Edar*^*downlessJ*^; Inc, incisors; M1, first molar; T1, first tooth; WT, wild type.

First, we looked at the dynamics of *Edar* expression in the *Edar*^*dlJ*^ mutant to check if R2 was indeed rescued in this mutant. This mutant encodes for a defective Edar protein because of a single amino acid change, but the gene is still transcribed. In contrast with the mutant epidermis, in which *Edar* expression stays at uniform low levels and hair fails to form (56), we still observe *Edar* restriction to tooth signaling centers (Fig 4A), consistent with teeth being formed. However, the dynamics of activation-inhibition mechanisms were modified in this mutant. We observed high variability between embryonic tooth rows, including left and right rows of the same embryo. This is in line with the high phenotypic variability seen in adults with mutations that affect the Eda pathway, in which two or three, or in rare cases, four, teeth of variable sizes are formed [53–55]. In the lower jaw (Fig 4A), we did not find obvious differences early in 12.5 dpc *Edar*^*dlJ−/−*^ embryos as compared with *Edar*^*dlJ+/+*^ embryos, all of them exhibiting restriction of *Edar* to the MS signaling center (S1 Fig). In both cases, wild type and mutant, *Edar* is next expressed in the whole dental epithelium (13.0 dpc, Fig 4A). However, no restriction was observed in *Edar*^*dlJ−/−*^ 13.5 dpc embryos as normally seen in their wild-type counterpart. As a matter of comparison, wild-type *Edar*^*dlJ+/+*^ embryos sampled with a body weight between 120 and 140 mg showed restricted *Edar* expression to R2, and 140–155 mg embryos showed restriction to R2 plus expression in the posterior, corresponding to the starting wave (S1 Table). In contrast, among the 26 *Edar*^*dlJ−/−*^ embryos sampled between 120 and 160 mg, all showed homogeneous expression in the dental epithelium. In fact, homogenous *Edar* expression was still observed in most 14.0 dpc *Edar*^*dlJ−/−*^ embryos at a time when homogenous *Edar* expression is again observed in wild-type *Edar*^*dlJ+/+*^ embryos (Fig 4A, S1 Table). From 14.5 dpc, a restriction to a signaling center we named T1 PEK (for Tooth 1 PEK) was observed in most *Edar*^*dlJ−/−*^ embryos, with or without expression in the "tail," while others still displayed more or less continuous *Edar* expression. We noticed that this signaling center in the mutant is found more posteriorly in the jaw than is the R2 signaling center (Fig 4B). At 15.0 dpc, we see either one signaling center with *Edar* expression in the tail or two signaling centers, named T1 and T2. Possibly, the latter case is due to approximately simultaneous patterning of two signaling centers from a dental epithelium that was showing continuous expression in the previous stage. At 15.5, T1 has developed into a tooth germ of variable size, from a very small one to a tooth germ equivalent in size to T2. To conclude, our results show that R2 patterning is both postponed and displaced posteriorly in the *Edar*^*dlJ−/−*^ mutant, and the resulting signaling center, T1, persists to form a tooth germ of variable size. This raises the question of whether R2 is rescued in the *Edar*^*dlJ−/−*^ context in which teeth are poorly grown.

We next used our model to explain this nonintuitive observation. Eda pathway loss of function has been shown to increase inhibition in teeth and other appendages [12,50], which is consistent here with the T1 signaling center being patterned further away from the anterior end of the dental epithelium. Therefore, we analyzed numerically the effect of a stronger inhibition, which is achieved in our mathematical setting by decreasing the rate of auto-inhibition, that is, by decreasing the strength of the negative autoregulatory feedback on the inhibitor. Interestingly, this was sufficient to recover qualitative behaviors consistent with the data: (1) T1 and T2 are formed later than R2 and M1 (horizontal red dashed line). (2) T1 is displaced posteriorly (vertical red dashed line). (3) The wave no longer destabilizes T1. Rather, a Turing pattern is formed that is not subjected to a palimpsest. These results suggest that a new wave of activation associated with the formation of the next signaling center will naturally destabilize any pre-existing signaling center, if inhibition from this pre-existing center is weak enough. In addition, our model does not impose any differences between signaling centers; more precisely, we do not impose that R2 is unstable or weak in essence. Rather, R2 is actively overwhelmed by the activation wave associated with M1 formation. This is a major output of our modeling effort but it is in contradiction with the current thinking, in which the R2 signaling center is considered to be autonomously unstable or weak, possibly due to its position near the diastema. Therefore, we decided to directly test this hypothesis.

### The anterior part of the dental epithelium (R2) is able to form a tooth if it is separated from the posterior part

**Fig 5:**
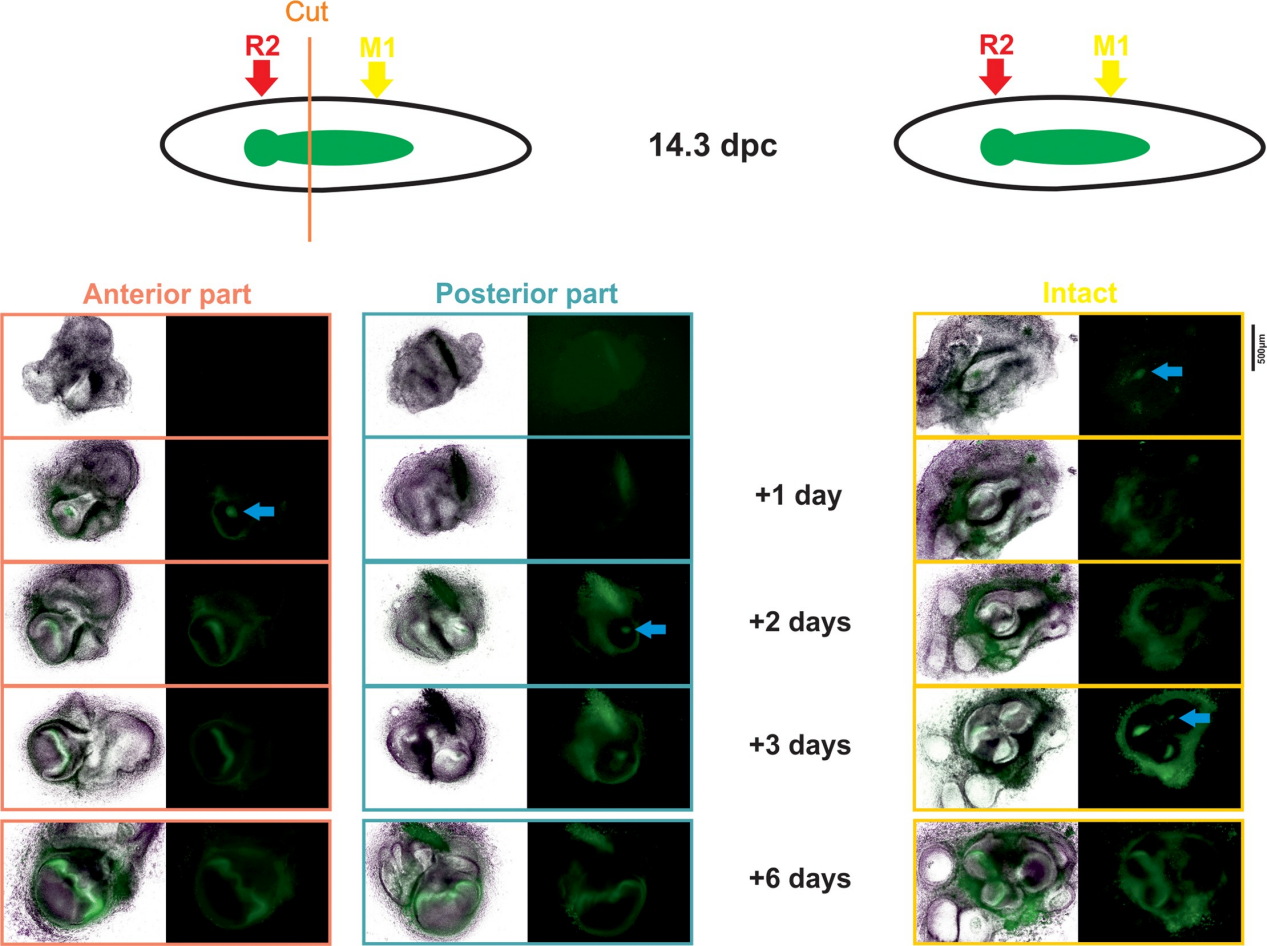
The anterior part of the molar field corresponding to the R2 signaling center can develop into a tooth germ when separated from the posterior part. The developing left and right molar regions were dissected from 14.3 dpc Shh-EGFP embryos according to the scheme and cultured for 6 days. Left panel: the most anterior part of the left molar region, comprising the R2 signaling center, was separated from the posterior part containing the early M1 signaling center and the posterior "tail." The following day, a signaling center was recovered in the anterior part and formed a tooth germ. After 2 days, a signaling center appeared in the posterior part, and this part formed a similarly sized tooth as the anterior part after 6 days. Right panel: the right molar region was cultured intact as a control. The second molar does not form before the third day. *Shh*-driven expression of EGFP in tooth signaling centers are indicated by a blue arrow. The experiment (culture of separated and control) was repeated 27 times with highly consistent results. dpc, days post coitum; EGFP, enhanced green fluorescent protein; M1, first molar.

If the anterior part of the dental epithelium is intrinsically defective for tooth formation, it should not be able to give rise to a fully developed tooth when removed from the early M1 signaling center. On the contrary, if the anterior part is not intrinsically defective but is normally overwhelmed by the M1-forming wave, as we suggest, a tooth should be able to develop from M2 when shielded from the influence of M1. To test this, we dissected the anterior part of the molar field, comprising R2, from the posterior part, comprising the early M1 signaling center and the tail. This experiment was performed in Shh–enhanced green fluorescent protein (EGFP) mice, where signaling centers are green fluorescent protein (GFP) positive. As expected from our predictions, the anterior part developed into a fully growing tooth. Remarkably, the timing of development was advanced compared with the posterior part by 1 day, in accordance with the R2 signaling center having been patterned earlier than the M1 one (Fig 5).

Taken together, these results are consistent with a model in which the R2 region is fully competent to form a tooth but is actively overwhelmed by the forming M1, resulting in the developmental palimpsest effect described.

### The formation of a large signaling center depends on Edar activity

We then focused on another feature of the mouse dental row, the incorporation of R2 into M1, which is hypothesized to play a crucial role for the formation of the anterior part of M1 both during development and evolution [33,36]. This corresponds to another curious behavior of *Edar* expression dynamics: the fusion of R2 and early M1 signaling centers soon after recovering from the palimpsest (Fig 3C).

**Fig 6:**
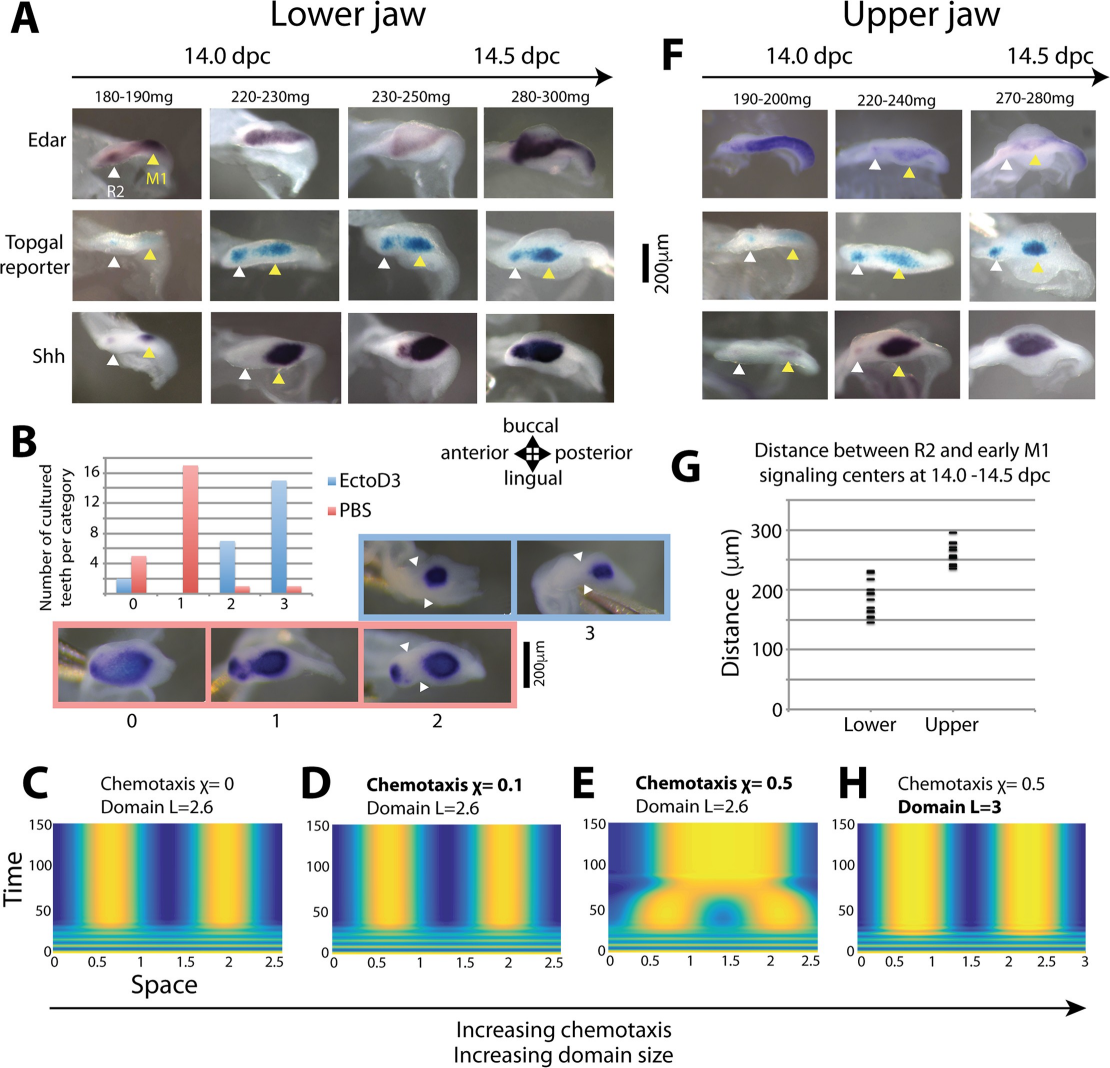
The formation of a large fused PEK depends on Edar signaling, possibly through chemotaxis. **(A,F)** Dynamics of *Edar* (upper) and *Shh* (lower) expression and TOPGAL reporter X-gal staining (middle) during the 14.0 to 14.5 dpc period when M1 PEK forms in lower (**A**) and upper (**F**) jaws of CD1 mice. In both jaws, *Edar* expression spans both the R2 and M1 regions before becoming restricted. In the upper jaw and very transiently in the lower jaw, expression restricts again to R2 plus the newly formed early M1 signaling center (220–240 mg and 180–190 mg, respectively). In the lower jaw, expression restricts to a large domain including both R2 and early M1 signaling centers (from 220–230 mg). In situ hybridization with *Edar* or *Shh* probes and X-gal stainings were performed on dissociated lower or upper jaw epithelia. The embryonic weights used to stage embryos are shown. Staining concentrated in R2 signaling centers is indicated with a white arrowhead, while early M1 signaling centers are indicated with a yellow arrowhead. **(B)** Lower molar rows were cultured from 13.0 dpc and, after 2 hours of recovery, they were treated for 40 hours with an Eda-blocking antibody (EctoD3) or with mock for 40 hours. The dissection process tends to interfere with the formation of the large signaling center that is normally seen in vivo (state 0): in most samples, low *Shh* expression is seen between R2 and early M1 signaling center (state 1), and some samples show signs of individual development of R2 (state 2); however, *Shh* expression in R2 is always maintained. In EctoD3-treated samples, R2 can further develop as a clear individual tooth bud (state 3). However, *Shh* expression in R2 is absent, and only a small posterior M1 signaling center is found. **(C, D, E, H**) Numerical simulation of a simple Turing model augmented with chemotaxis in a fixed, matured domain with a simple configuration consisting in two Turing peaks. **(C)** In the absence of chemotaxis (χ = 0), two Turing peaks form in a domain of length L = 2.6. (**D, E)** In a model in which the activator stimulates the production of a chemoattractant (over a certain threshold), Turing peaks form first, and later, chemotaxis fuses both peaks in a single large peak (χ = 0.5, E). However, fusion does not happen with insufficient activity of the chemoattractant (χ = 0.1, D). (**H)** On a larger domain (L = 3), when a Turing pattern with a larger wavelength is chosen, levels of chemotaxis identical to those in E (χ = 0.5) are no longer sufficient to fuse the peaks. **(G**) Distances between R2 and M1 signaling centers, as measured with TOPGAL reporter on dissociated epithelia at 14.0 dpc in the lower and upper jaws (*n* = 26 for lower jaw, *n* = 24 for upper jaw; embryonic body weight comprised between 230 and 320 mg). R2 and M1 signaling centers are closer in the lower jaw than in the upper jaw. dpc, days post coitum; M1, first molar; PBS, phosphate-buffer saline; PEK, primary enamel knot.

We examined in detail the 13.5–14.5 dpc period, which corresponds to M1 PEK formation. To do so, in parallel with the dynamics of *Edar* expression, we monitored Shh and Wnt pathway activity (the latter with the TOPGAL reporter), which are recognized markers of tooth signaling centers (Fig 6A). In late 13.5 dpc/early 14.0 dpc embryos, *Shh* expression and TOPGAL X-gal staining reveal that the early M1 signaling center starts to form. Some faint *Shh* expression is occasionally seen in R2 while X-gal staining persists there, presumably in part due to the long half-life of the B-galactosidase. At this stage, *Edar* is also focused in R2 and early M1 signaling centers, yet low expression can also be seen around. In slightly older embryos, robust *Edar* expression is seen in a domain spanning both signaling centers and is aligned with the barely formed cervical loops. This *Edar* expression is followed by anterior expansion of *Shh* expression, which finally spans the position of former R2 and early M1 signaling centers. We also observed up-regulation of TOPGAL activity during the same period. Altogether, these results show that R2 and early M1 signaling centers are repatterned as a single large signaling center highlighted by *Edar* expression. This early event prefigures TOPGAL activity and *Shh* expression relocalization into a large signaling center.

Because *Edar* expression prefigures the anterior expansion of the *Shh* expression domain and *Edar* has been shown to regulate *Shh* [45,56,57], we wanted to test if Edar signaling is necessary for anterior expansion and the formation of a large M1 PEK. To specifically test this, we dissected 13.0 dpc lower molar regions, when R2 has already formed, and cultured them for 40 hours with or without an interfering antibody, so that we knocked down Edar signaling in the next period of M1 PEK formation. We then visualized *Shh* expression on isolated epithelia. In untreated samples, we occasionally observed a large M1 PEK similar to in vivo samples (Fig 6B, state 0), but most often it was split into a R2 spot and an enlarged early M1 spot bridged by a narrow domain of *Shh* expression (Fig 6B, state 1). This can be explained by the fact that the dissection process could change the activation-inhibition balance in favor of inhibition (as proposed in [58]), which, according to the predictions of our model, should favor R2 persistence. In treated samples, we mostly recover a small, very posterior signaling center, which corresponds to early M1 *Shh* expression (Fig 6B, state 2 and 3). Moreover, the dental epithelium is morphologically different, always showing a bud, followed by a small cap and a "tail" (states 2 and 3). Thus, in the absence of Edar signaling, *Shh* expression is lost in the R2 region, and only a small PEK forms that is equivalent in size and position to the early M1 signaling center, and which drives cap transition there. Taken together, our results show that Edar signaling is essential for the formation of a large PEK that encompasses R2 and early M1 signaling centers.

### Coupling chemotaxis to the Turing system reproduces biological variability in the fusion of signaling centers

Recent studies have pointed out that chemotaxis may play a role in the formation of tooth and hair placodes [11,48,49] and that the Eda pathway activated centripetal migration in the placodal epithelium (45). We noticed that the TOPGAL stainings tend to contract in the anteroposterior direction as the M1 signaling center matures and the distance between R2 and early M1 signaling center decreases (compare 14.0 and 14.5 dpc samples in Fig 6A). This suggests that cell movements may take part in the formation of the large signaling center.

To evaluate this possibility, we incorporated cell motion through chemotaxis in a simple Turing system producing two peaks, thus starting with the situation when R2 and M1 signaling centers coexist (Fig 6C). We assumed that the chemoattractant pattern corresponds to the activator pattern so that cells move towards regions of higher activator concentration. Cell aggregation mediated by chemotaxis requires a positive feedback loop between cell density and chemoattractant concentration [19–20]. In our setting, without the addition of extra molecular entities, this feedback loop can act either directly on the activator-chemoattractant, as in the classical Keller-Segel system [20], or via down-regulation of the inhibitor, if a higher cell density negatively affects inhibitor concentration. The latter configuration produced the expected behavior: signaling centers first form, and then they fuse into a single large signaling center (Fig 6E). This is consistent with the intuitive idea that fusion requires sufficiently long-range communication between the two Turing peaks and thus a feedback on the long-range diffusing species, which in our case is the inhibitor. Reducing chemotaxis efficiency prevented fusion, consistent with our experiments, in which we reduce Edar signaling, and presumably, chemotaxis (Fig 6D).

Nonetheless, adding chemotaxis to our system does not automatically result in fusion into a single spot. In our simulations, we observed that the transition between the absence of fusion and fusion depends on various parameters. The reason is that the activator-chemoattractant has a direct positive effect on the inhibitor but an indirect negative effect on the same inhibitor by means of cell recruitment. These ambivalent effects make chemotaxis able to compensate the segregation due to Turing patterning in some situations or reinforce it in other situations. For example, we observed that chemotaxis can favor pattern formation when the Turing system fails to produce a pattern alone. This suggests that chemotaxis may be part of the normal formation of tooth signaling centers, even when they stay separated.

In line with this, we noticed that there is no fusion in the upper jaw (Fig 6F), where the distance between signaling centers is initially larger by about 30% (Fig 6F and 6G). In the model, this small increase of the domain size increases the Turing wavelength and is sufficient to abolish the fusion (Fig 6E and 6H). With our parameter settings, a 15% increase in domain size could prevent fusion under the same chemotaxis efficiency.

In conclusion, the nontrivial interaction between chemotaxis and a Turing system appears to be a plausible mechanism to explain the variability in the dynamics of tooth signaling centers between the lower jaw and the upper jaw of the wild type, or between the wild type and an *Edar* loss of function.

## Discussion

In this study, we have studied the highly complex and dynamic behavior of signaling centers responsible for tooth patterning in the mouse jaw. Patterning is usually seen as a directional process rather than a dynamic process that could take circuitous routes. However, we show that patterning of the first molar involves what we called a developmental palimpsest, where patterns are established, erased, rescued, and fused to give rise to new patterns. Using a mathematical approach, we show that these behaviors, despite seeming to be complex, can be produced by the activity of simple mechanisms: a Turing pair with two regimes, as well as chemotaxis.

### From similarities in *Edar* expression dynamics to differences between hair and tooth patterning

In this study, we have revealed the highly dynamic expression of *Edar* in the developing molar row. This dynamic is superficially similar to that seen during hair patterning. This is not surprising because hair and tooth patterning share many common features [29,59], making their comparison very instructive. Below, we compare these two systems in light of our results.

In teeth, as in hair, *Edar* expression becomes restricted to the signaling center as it is patterned. We have noticed, however, two substantial differences: (1) in skin, the initial basal levels of *Edar* are up-regulated in the placode and down-regulated in its vicinity. This is thought to be pivotal for placode patterning, in which Eda signaling is necessary to stabilize and refine an otherwise labile Wnt-dependent placode prepattern [12,46]. In the molar field, *Edar* expression in the dental epithelium reaches levels pretty similar to restricted expression in the signaling center, suggesting that *Edar* is actively stimulated in both cases. This up-regulation may rely on ActivinβA, which stimulates *Edar* expression in tooth cultures [47], and on the Wnt pathway, which plays a central role in tooth formation and is involved in *Edar* basal expression in hair [12,46]). Down-regulation may rely on the BMP pathway, as is the cases in hair [12,46]. (2) We show that this regulation still occurs in the *Edar* mutant, a major difference with hair, for which the lack of Edar signaling freezes the initial state of uniform basal levels of *Edar* expression [12]. Self-activation of the pathway thus plays a more minor role, if any, in teeth.

We believe that these differences on *Edar* regulation may reflect differences in the balance of the different processes participating in hair and tooth formation. For the formation of hair placodes, Turing-like mechanisms establish a noisy prepattern, with local sources of FGF signaling. Mesenchyme condensation towards these sources then refines and reinforces the pattern [11]. In mutant conditions that remove the epithelium prepattern, it appears that the mesenchyme can still produce spaced foci of cell condensates [11]. Thus, the mesenchyme is also capable of autonomous self-organization, but this capacity is not fully exploited because of the prepattern imposed by the epithelium.

The formation of tooth signaling centers seems to rely on a different equilibrium between the two tissues. The formation of a PEK is highly dependent on mesenchyme condensation, as seen in bud-arrested tooth germs in which condensation fails [28]. Modeling the gene network of epithelium–mesenchyme interactions in teeth also led to the suggestion that both tissues work in concert, rather than one dominating the other [27]. These intrinsic differences may explain why *Edar* loss of function abolishes pattern formation in the epithelium-dominated context of hair formation but only results in spatiotemporal modifications in the more balanced context of tooth formation.

### A model for sequential patterning of signaling centers in the dental epithelium

In this study, we assume that the complex spatiotemporal changes in *Edar* expression highlight waves of activation in the dental epithelium. Each of these waves results in the patterning of a signaling center, and they are reiterated upon posterior growth of the dental epithelium. We note that this growth zone could be the *Sox2*-positive region shown in [24]. We built an RD mathematical model to describe this behavior. In this macroscopic model, molecules are treated as a continuum and set on a one-dimensional space to model the anteroposterior dimension of molar row formation. We also chose to consider only two types of Turing in-phase molecules, corresponding to an activator and an inhibitor. This is, of course, a high level of abstraction. Tooth genetics has revealed many molecules from the epithelium or the mesenchyme that could participate in the activation-inhibition mechanisms (with both in-phase and out-of-phase patterns). Studies generally choose to focus on only some of them, for the sake of simplicity. For example, Cho and colleagues [31] have shown that perturbation of Shh function releases inhibition on the next forming tooth and, moreover, up-regulates Wnt and FGF pathways and down-regulates Sostdc1. They then showed that it is theoretically possible to generate a Turing pattern in a distally growing field from such three species: an activator, an inhibitor, and a mediator allowing the negative feedback between the activator and the inhibitor. From this theoretical result, they suggested that Wnt, Sostdc1, and Shh could play these respective roles to pattern teeth. O’Connell and colleagues focused their work on cross-regulatory relationships between Wnt and BMP signaling [27]. Here, it was not our purpose to identify these molecules. Instead, our modeling effort aimed at providing a theoretical framework for sequential tooth formation, with a special focus on the spatiotemporal dynamics of the system. Moreover, although our model explicitly aims to describe activation in the epithelium (*Edar* dynamics), this does not mean that the activator-inhibitor couple in our model should be seen as an abstraction for Turing reactions in the epithelium only. We do not rule out that our model could synthesize the activation-inhibition reactions arising from epithelia-mesenchymal interactions and giving rise to the Turing pattern. For example, mesenchymal inhibitors such as Sostdc1 could take their part in the inhibition.

One key ingredient in our model is the transition from a bistable regime to a Turing regime, which is facilitated by the structural similarity between both regimes in our activator-inhibitor system. There are many ways to achieve this transition. We opted for decreasing the rate of auto-inhibition of the inhibitor as tissue maturates. However, we believe that our conclusions hold true for various ways of modifying the activator-inhibitor dynamics, provided that the reaction terms follow the picture depicted in S1 Fig. For instance, increasing the activation rate from the activator onto the inhibitor would act similarly. Dedicated experiments would ideally confirm that tissue maturation has an impact on the regulation feedback rates. We note that the maturation-induced modification of RD parameters is not specific to our model. For example, a study by Prochazkova and colleagues [60] proposed that diffusion rates (rather than feedback rates) change upon differentiation of tongue papillae, influencing their final size.

A second originality of our model came from modeling the observation that new waves of *Edar* expression repeatedly initiate posteriorly and can wash out the focused *Edar* expression in the rudimentary signaling centers. This was achieved by means of a bistable wave, which initiates on the posterior side after a long delay of sustained down-regulation. We found that this wave, when interacting with a Turing pattern already established on the anterior side, can destabilize and even erase it. The fate of the Turing mature pattern depends on the details of the model, through the intensity of the feedback regulations as well as the timescales. This spatiotemporal interaction between a Turing pattern and an RD wave is, as far as we know, original.

Our model explicitly assumes that the mesenchyme is responsible for periodic activation priming of the newly grown epithelium. This dependence is consistent with a body of evidence showing that mesenchyme activity is necessary for the induction of PEK formation and sequential tooth formation [27,30,61]. We also know that mesenchyme activity depends on the Msx1-Bmp4 feedback loop [62–64], which is itself dependent on a mechanical signal provided by mesenchyme condensation [28]. When this loop is defective, sequential tooth formation can stop at different stages, from no tooth forming, only one, or only two instead of three [63,64]. It can also simply stall until adequate levels of Bmp4 signaling are reached, as seen in the *Barx1* mutant [65]. The mesenchymal Bmp4 signal is part of a Wnt-Bmp regulatory network whose integration drives signaling center formation [27]. It is also known that the mesenchyme produces ActivinβA, a potent inducer of both tooth formation [30] and *Edar* expression. In the absence of further knowledge about how the mesenchyme could prime the waves of activation observed in the epithelium, we introduced in our model an extrinsic component representing the interaction with the mesenchyme, and chose a parsimonious way to provide it with an oscillatory behavior. For this, we assumed that the mesenchyme activity is stimulated by the activator and feeds back on it in the newly grown area. Below a certain threshold, it will act to increase the concentration of the activator. Above a certain threshold, it will act to decrease the concentration of the activator. This is the main limitation of our current model, which raises the very interesting question of what the mechanisms enabling periodicity in the molar row are. In particular, experimental approaches will be needed to determine if the mesenchyme oscillates and how these oscillations relate to the *Edar* waves in the epithelium. Can the mesenchyme oscillate on its own, or are oscillations a property of the epithelium-mesenchyme cross-talk along the anteroposterior axis? For example, one possibility suggested by our model would be that the posterior mesenchyme self-activates when it has grown enough, because it is then far enough from the inhibitory influence of an epithelial signaling center. This could be investigated by cutting experiments that would abruptly remove this inhibitory influence.

Despite this limitation, we note that our current model exhibits a relevant feature, because inhibition from the Turing spot locks the bistable system of the newly grown epithelium in the “no activation” state. This means that, in the absence of a wave of activation triggered by the mesenchyme, sequential addition will stop, in contrast with a standard Turing system in a growing field. This is consistent with mutants in the Bmp4-Msx1 axis, in which sequential addition stops after M1 or M2 formation.

Although our modeling approach mainly focuses on qualitative insights, we wanted to assess the robustness of pattern formation and developmental palimpsest in our model. We found a suitable model parametrization and tested its sensitivity with respect to patterning (see S1 Text for details). Although the results are generally robust enough to moderate parameter changes (10%–50%), it is interesting that the developmental palimpsest can be abolished in many ways, changing auto-inhibition but also temporal dynamics and synchronicity between events. This is consistent with the marked tendency of molar row development towards supplementary molar formation: it can happen in mutants of many different pathways; moreover, it often occurs without major changes in other aspects of tooth development.

### Comparison with models of sequential patterning in other organs

Our model shares some similarities with models of somitogenesis. First of all, almost all somitogenesis models include a clock driving gene expression oscillations, forming traveling waves moving through the tissue [66]. Even cells isolated from the presomitic mesoderm exhibit oscillations [67]. However, whether such a bona fide molecular oscillator will be found in the tooth system remains an open question. We note that tissue-scale oscillations have been observed in limbs, whose development shares similarities with that of epithelial appendages including teeth [68]. We also envision other possibilities relying on tissue properties rather than cell properties, for example, emerging from the cross-talk between the epithelium and the mesenchyme, as suggested above.

Second, in the long-prevailing models of somitogenesis, the clock is combined with a gradient of Fgf/Wnt signaling that maintains the oscillations in the posterior part and determines the position where the traveling wave is frozen into a stationary pattern, which will define somite boundaries [69]. Our model does not comprise such positional information. In a more recent model of somitogenesis, traveling wave and pattern formation are produced by a Turing pair with a nondiffusing activator and a diffusing inhibitor [15]. Pattern formation arises when the traveling wave breaks next to the previously formed stripe (which acts as a stable source of inhibitor), and local interactions in this region promote activator increase to form a new Turing stripe in the vicinity of the previous stripe. This model shares an obvious similarity with our model: a Turing pair exhibits different behaviors along the anteroposterior axis. It can be oscillations with traveling wave and Turing in the Cotterel model, versus bistable with traveling wave and Turing in our model. However, the switch between both behaviors arises as a local emergent property next to previously formed stripes in the Cotterel model, whereas it is explicitly introduced in our model as a result of maturation. Moreover, in our model, the oscillations are provided by an exogenous oscillator at the posterior boundary, fulfilling the function of the mesenchyme. We acknowledge that the Cotterel model might apply to the tooth system, and it will be interesting to test if the palimpsest can be obtained with such a model.

Our study also shares superficial similarities with the sequential patterning of feathers. In this system, a priming wave of activation is observed in the epithelium, giving rise to a stripe in the back of the chick embryo, which is then broken into a spot pattern giving rise to individual feathers. Pattern formation in the model by Painter and colleagues relies on chemotaxis rather than RD [22]: moderate cell aggregation drives stripe formation in the primed epithelium through an FGF-dependent positive feedback, and strong local aggregation introduces a BMP-dependent negative feedback that contributes to breaking the stripe into spots. The behaviors of the two systems are similar: the broad *Edar* expression could be compared to the priming wave or the first stripe, and the formation of the signaling centers could be compared to the breaking of the stripe into spots. These models also converge conceptually. Stripe formation in the feather model and *Edar* activation wave in the tooth model mainly rely on positive feedback. Spot formation and signaling center formation both rely on the introduction of a sharper negative feedback. We take this as an indication that this sequence of activation might be a general property of epithelial appendages (feathers, hair, teeth) that can be captured by very different, nonexhaustive models. We also want to stress that our model is meant to recapitulate local activation/long-range inhibition mechanisms rather than specifically RD mechanisms, and we do not exclude that the biological mechanisms it captures are based on chemotaxis, as in the Painter model.

### Making and erasing patterns: A developmental palimpsest characterizes formation of the first molar

Lastly, we would like to emphasize how the current model successfully recapitulates a number of counterintuitive behaviors of the system and informs us on the possible underlying mechanisms.

Previous studies had already revealed several complex behaviors in the growing dental epithelium: (1) the transient patterns of MS and R2 signaling centers, supposedly vestiges of premolar signaling centers; (2) the rescue of an abortive tooth germ, R2, in a large number of genetic conditions; (3) the transient coexistence of R2 and early M1 signaling center, followed by their fusion in a large signaling center in the lower jaw.

The present data and our simple model suggest that these complex behaviors are the fruit of rather simple but highly dynamic interactions in the growing tooth field.

As viewed from *Edar* expression, the pattern constituted by MS and, later, R2 signaling centers is erased to give rise to a second wave of patterning, illustrated by a broad *Edar* expression in the dental epithelium at respectively 12.5–13.0 dpc and 13.5–14.0 dpc. This was recapitulated in the model by enabling the bistable domain to form a traveling wave that can destabilize a previously formed signaling center, if inhibition in the latter is not too strong. Aside from recapitulating *Edar* expression, the traveling wave has more profound implications. Indeed, it implies a first paradigm shift that vestigial buds are not committed to abort as usually thought, for example, due to their proximity with the diastema, thought to serve as a source of inhibitors [70,71], or through the expression of specific molecules [72]. Rather, or on top of that, functional signaling centers are actively competed by the next round of activation as the dental epithelium grows. Such a balance explains why the anterior part of the molar row is very sensitive to environmental perturbations, such as the dissection associated with tooth culture and to genetic perturbations, as many of them result in supplementary tooth formation there. It also explains why even conditions that produce a more inhibitory context than the wild type can produce a supplementary tooth. Indeed, our model predicts that if inhibition is increased, as it is commonly assumed to be in mutants of the Eda pathway, then the Turing pattern can still form yet with a slightly longer wavelength, but the traveling wave is almost immediately suppressed. This is exactly what we document in *Edar*^*dlJ*^ mutants for the R2 signaling center: it forms more posteriorly, and we see no traveling wave that would erase it. Rather, it persists to form a tooth bud. Our tooth culture experiment with the anterior part of the molar field comprising the R2 signaling center demonstrates that R2 has the potential to fully form a tooth when not actively competed by the M1 signaling center in the wild-type situation. Consistent with our results, Li and colleagues reported that FGF8 application could rescue tooth germ development in the mouse diastema only when it was separated from the molar and incisor buds [73]. In conclusion, our results extend the prevailing model of Kavanagh and colleagues [30], in which inhibition between forming teeth is unidirectional (from M1 to M2, to M3), by showing that inhibition can be bidirectional and subtly dependent on the temporal dynamics of the system.

In the wild type, after broad *Edar* activation at 13.5–14.0, a new pattern of *Edar* restriction forms that is markedly different between the lower and upper jaws. In the lower jaw, independent R2 signaling center and early M1 signaling center are seen either very transiently when looking at *Edar* expression or for a longer time when looking at *Shh* expression or TOPGAL activity. These centers are then rapidly fused into a single elongated signaling center. In the upper jaw, *Edar* restricts to the R2 and M1 signaling centers and remains as such. Therefore, the palimpsest is observed in both jaws, enabling the co-occurrence of two signaling centers, but only in the lower jaw does some additional mechanism enable their fusion into a large signaling center. Here, we introduced chemotaxis to the model because it has been involved in hair placode formation, both at the level of the epithelium [48,49] and the mesenchyme [11,22]. This was sufficient to recapitulate a number of interesting features: (1) chemotaxis changes the RD so that the system first makes two peaks that later fuse into a single, larger peak. This is reminiscent of the large M1 signaling center. (2) This behavior is sensitive to the distance between the initial peaks, and a 15% increase was sufficient to impede fusion. The measured 30% difference between the R2–M1 distance in the lower jaw, where fusion occurs, and the R2–M1 distance in the upper jaw, where fusion does not occur, may thus be sufficient to explain the difference in fate in the two jaws. (3) Finally, reducing chemotaxis was sufficient to impede fusion. This may explain why, in our culture system, inhibition of Edar activity impedes fusion, although the distance between R2 and M1 does not seem drastically changed. These roles for chemotaxis would require experimental confirmation in our system: This could be tested, for example, by manipulating cultures of tooth germs with drugs impeding actin-based cell movements, as in [48].

Interestingly, we show that chemotaxis plays an ambivalent role in our model. Depending on the conditions, it acts in favor or against the Turing pattern, or it can be relatively neutral. We propose that this ambivalent role contributes to explaining the versatility of this biological system, with regard to genetic and environmental perturbations.

### Conclusion

An important lesson from the tooth system is that patterning events may be less straightforward than usually thought, and patterns may be dynamically drawn and erased or refined during embryogenesis. In other words, developmental palimpsests may be a common feature. One reason for this is historical. Systems are the product of evolution, and as pointed out by F. Jacob, evolution proceeds as a tinkerer, not as an engineer [74]. Extant patterning mechanisms are thus modified versions of ancestral mechanisms, not purposely designed from scratch. Our study is consistent with recent studies on the fine-scaled temporal dynamics of gap gene patterns in dipterans (e.g., a progressive anterior shift of the gap genes pattern). As shown here for the *Edar* mutant, incorporating these dynamics into models provided a better explanation for mutant phenotypes [9,75,76]. Moreover, these curious dynamics are also likely the vestige of an ancestral mode of segmentation [75,77]. In summary, we believe these two systems illustrate that temporal dynamics of developmental systems needs to be studied and, moreover, to be studied in the light of evolution to fully explain how the system reacts to perturbations. Indeed, embryonic patterns can be highly dynamic, and thus dynamics can be essential to the outcome of the patterning process.

## Materials and methods

### Ethics statement

Housing of animals and animal experimentations were conducted under animal care procedures in strict accordance with the guidelines set by the European Community Council Directives (2010/63/UE) as well as with the national guidelines (ID 39/2009). All mice used in this study were killed by cervical dislocation. Experimental procedures relative to Figs 1, 3, 4 and 6 were approved by the Institutional Animal Care and Use Committee CECCAPP, Lyon, France (# ENS-2009-027 et # ENS-2012-046). Experimental procedures relative to Fig 5 were performed under supervision of the Professional committee for guarantee of good life-conditions of experimental animals at the Institute of Experimental Medicine, the Czech Academy of Sciences, Prague, Czech Republic, and approved by the Expert Committee at the Academy of Sciences of the Czech Republic (permit number: 81/2017).

### Mouse breeding and embryo harvesting and staging

CD1 mice were purchased from Charles River (Germany and France). Other mice have been bred at the PBES (Lyon, France). TOPGAL mice (Tg(Fos-lacZ)34Efu) carrying three LEF1/TCF1 binding sites fused to a minimal *c-fos* promoter driving *lacZ* expression were backcrossed against CD1 mice for 10 generations [78]. TOPGAL positive mice were screened by standard lacZ staining performed on the first phalange cut from PN4-PN7 newborns. The *Edar*^dl-J^ mice (FVB background) were obtained from Denis Headon. They carry a G to A transition mutation causing a glutamate to lysine substitution in the death domain of the Edar protein (E379K [79]). The strain was maintained by crossing heterozygotes with homozygotes, and wild-type and *Edar*^dl-J/dl-J^ mice used in experiments were derived from this same stock.

In order to harvest embryos every 12 hours of development, mice were kept under two different day-night light cycles. Mice were mated overnight and vaginal plugs were detected the next morning, noon being indicated as the embryonic day (ED) 0.5. Pregnant mice were killed by cervical dislocation and embryos were harvested and weighted as described earlier [33,80].

For cut teeth culture experiments, CD1 females were crossed with males carrying the fusion protein *Shh*-EGFP and Cre recombinase from the endogenous *Shh* locus (B6.Cg-*Shh*^*tm1(EGFP/cre)Cjt*^/J [81]), which enabled determination of *Shh* expression using fluorescence. The breeding pairs B6.Cg-*Shh*^*tm1(EGFP/cre)Cjt*^/J were purchased from the Jackson Laboratory (Maine). Mice were genotyped using the Jackson Laboratory’s protocols.

### Mandible epithelium dissociations

Mandibles and maxilla were dissected in Hank’s medium and treated with 10mg/mL Dispase II (Roche) at 37°C for 1 hour to 2 hours 20 minutes depending on the embryonic stage. Epithelium was carefully peeled and fixed in 4% PFA.

### Whole mount in situ hybridization and X-gal staining

Embryonic mandibles, maxilla, or dissociated epithelia were fixed in 4% PFA solution overnight at 4°C and in situ hybridization was done according to a standard protocol. DIG RNA probes were transcribed in vitro from plasmids described elsewhere: *Shh* [82], *Edar* [47]. TOPGAL embryonic mandibles or dissociated epithelia were fixed in 4% PFA for 15 minutes only and stained with X-gal according to a standard protocol. Samples were documented on a Zeiss LUMAR stereomicroscope with a CCD CoolSNAP camera (PLATIM, IFR128, Lyon) or on a LEICA MFA205 stereomicroscope with a DFC450 camera (IGFL, Lyon).

### Organotypic culture and treatments

The lower molar region of 13.0 embryos were dissected and cultured according to methods described in [30]. Dissection was made carefully reproducible in terms of mesenchyme quantity left around the tooth germ. In order to control for possible developmental stage effects, embryo weight of each tooth germ was recorded, and one tooth germ was used as a control, while its contralateral tooth germ was used for treatment. Following a period of 2 hours of recovery, the medium was changed for a new medium supplemented with 5μg/mL of a function-blocking anti-Eda antibody (ectoD3 [83]). Tooth culture was stopped at 40 hours (S2 Fig) and epithelia were dissociated for 15–30 minutes with 10 mg/mL Dispase II (Roche) at 37°C.

### In vitro cultures of anterior and posterior parts of M1 tooth primordium

Left and right M1 tooth germs of ShhEGFP^+^ mouse embryos at 14.3 dpc were dissected identically from the embryonic lower jaw. In particular, one paid attention that the amount of the mesenchyme was almost identical and it should influence left and right M1 germs in the same way. The left M1 germ was then cut into anterior and posterior parts (for details, see Fig 5). Both parts were cultured separately on PET track–etched membrane according to the method described previously in [84]. Contralateral intact M1 dissected tooth germ from the same specimen was used as control. Cultures were photographed daily using an inverted fluorescent microscope Leica AF6000 (Leica Microsystems GmbH, Germany), from the day of dissection up to day 6 of culture.

### Mathematical modeling

The model and the parameters are described in S1 Text. The code written in matlab can be downloaded from https://github.com/monikatwarogowska/teeth-patterning-simulator. In order to run on of the six simulations described in the article, it is sufficient to run the main file (teethsolver.m).

## Supporting information

S1 Text A detailed description of the model, together with simulations under different parameter ranges. (PDF)

S1 Table A table describing the *Edar^dlJ−/−^* samples supporting Fig 4. *Edar*^*DlJ*^, *Edar*^*DownlessJ*^.
(XLSX)

S1 Fig Edar expression in 12.5 dpc *EdardlJ^−/−^* embryos is similar to wild type. dpc, days post coitum; *Edar*^*dlJ*^, *Edar*^*downlessJ*^.
(JPG)

S2 Fig Pictures showing ectoD3 and control cultures after 40 hours. (TIF)

S1 Movie A movie of the simulation shown in Fig 2. The evolution of activator concentration (red), inhibitor concentration (blue), and domain maturation (green) is shown as the domain grows.
(GIF)

S2 Movie A movie of the simulation shown in Fig 3. The evolution of activator concentration (red), inhibitor concentration (blue), and domain maturation (green) is shown as the domain grows.
(GIF)

S3 Movie A movie of the simulation shown in Fig 4. The evolution of activator concentration (red), inhibitor concentration (blue), and domain maturation (green) is shown as the domain grows.
(GIF)

## Abbreviations

dpc: days post coïtum
ED: embryonic day
Edar^dlJ^: *Edar*^*downlessJ*^
EGFP: enhanced green fluorescent protein
GFP: green fluorescent protein
M1: first molar
M2: second molar
M3: third molar
MS: mesial segment
PEK: primary enamel knot
RD: reaction–diffusion
R2: rudiment 2
