# Supplementary material for: Modeling Edar expression reveals the hidden dynamics of tooth signaling center patterning
Source: PLoS Biol. 2019 Feb 7;17(2):e3000064. doi: 10.1371/journal.pbio.3000064 (PMC6382175; doi:10.1371/journal.pbio.3000064)
Supplement: S1 Text — (PDF) [file pbio.3000064.s001.pdf]

SUPPLEMENTARY MATERIAL

S1 Mathematical model of teeth patterning

We introduce a mathematical model of reaction-diffusion type, which describes concentrations of a short range activator and a long range inhibitor on a one dimensional growing domain.

The rationale behind our model is to the observation that Turing patterns and traveling wave propagation can arise from the same system of reaction-diffusion equations, up to the change of one parameter. As the domain of interest has different levels of maturation across the epithelium – immature in the newly grown tissues versus mature in the old area – we propose that maturation allows for a switch between a bi-stable system (wave-like patterns) in the immature area versus a stable system (Turing-like pattern) in the mature area. On top of that, we postulate that maturation itself is enhanced by the activator.

Traveling waves are initiated in the distal part of the dental epithelium and then travel towards the anterior region. We propose an oscillatory system localized in the distal area to model sequential bursts of activator up-regulation.

It is an outcome of the model that the time-sequential bursts of distal up-regulation are transformed into a spatial sequence of up-regulated signaling centers (SC).

The possible fusion of SC is considered as a secondary effect, possibly mediated by chemotaxis signaling and cell migration.

S1.1 Mathematical setting: reaction-diffusion equation on a growing domain

Let $\Omega(t) = [0, r(t)]$ be a growing one-dimensional domain corresponding to the anterio-posterior axis of the dental epithelium. Time evolution of the concentration $\rho = \rho(\xi, t)$ at point $\xi \in \Omega(t)$ and time $t > 0$ of a reacting and diffusing substance can be described by

$$\partial_t \rho + \partial_\xi (v\rho) = D\partial_{\xi\xi}\rho + f, \tag{S1}$$

where $D > 0$ is the diffusion coefficient and $f = f(\rho, \xi, t)$ defines reaction kinetics. The growth of the domain generates a velocity field $v(\xi, t)$ that appears in the equation as a transport term "$\partial_\xi(v\rho)$" [9, 3]. For the sake of simplicity, we consider an apical domain growth, which means that the velocity field is linear in the rightmost interval $I(t) = [r(t) - \Delta_r, r(t)]$, with $\Delta_r > 0$,

$$v(\xi, t) = \begin{cases} \lambda_G(\xi - (r - \Delta_r)) & \text{if} \quad \xi \in I(t) \\ 0 & \text{otherwise} \end{cases}, \tag{S2}$$

where λ_G is a positive, constant growth rate. We assume $\Delta_r \ll r(0)$, which leads to a free boundary problem with the following explicit domain size

$$r(t) = r(0) + \lambda_G(t - t(0)). \tag{S3}$$

The problem can be reformulated on an interval of fixed unit length by the following change of variables

$$\xi \mapsto x = \frac{\xi}{r(t)} \in [0, 1]. \tag{S4}$$

Using the assumption (S3) equation (S1) becomes

$$\boxed{\partial_t \rho = \frac{D}{r^2(t)} \partial_{xx} \rho + x \frac{\dot{r}(t)}{r(t)} \partial_x \rho + f,} \tag{S5}$$

where $\dot{r}$ denotes the derivative of r with respect to time.

S1.2 Activator-inhibitor system of teeth patterning

In this section we describe a mathematical model of teeth patterning, which produces numerical results presented in the main part of the article. It is based on the following assumptions:

- Patterning is governed by the interaction between a generic activator and its inhibitor, which represent gene-encoded proteins. Reaction kinetics cover two regimes: a *Turing regime*, in which the system can evolve to a stationary heterogeneous pattern [12, 6], and a *bi-stable regime*, in which the system develops naturally moving interfaces that can make dynamic transitions from one state to another. Compared to feather and hair, much less is known about exact molecular entities underlying the activator-inhibitor mechanism in teeth development. So we opt for generic activator-inhibitor reaction kinetics that are commonly used in pattern formation modeling.

- Epithelial tissue growth is restricted to a small region on the distal part. Furthermore, epithelium is characterized by its level of maturation, which we use as a switch between the *bi-stable regime* (low maturation) and the *Turing regime*. Since the *Turing regime* and the *bi-stable regime* are conceptually close between each other, the switch is dependent upon a single parameter in our system. This change between regimes of the system is motivated by the experimental data showing that signaling centers develop in the old part of the tissue (mature), whereas new bursts of activator arise on the newly grown region (immature).

- An exogenous oscillating system is responsible for periodic bursts of activator localized at the rightmost tip. These bursts are necessary for the periodic up-regulation of activator in the immature region, as observed in our experiments. Local up-regulation in a bi-stable system may result in the propagation of wave as it is well-known in the theory of reaction-diffusion equations [1]. We hypothesize that this oscillation could be regulated by epithelium-mesenchyme interactions, although we do not explicitly encodes this coupling in the model as we believe it has a minor impact on our study.

S1.2.1 Activator-inhibitor system

Our model describes concentrations of the activator a and the inhibitor i by a system of reaction-diffusion equations on a growing domain, which after rescaling to a unit interval (S5) write as

$$\partial_t i = D_i r^{-2}\partial_{xx} i + x\phi(r)\partial_x i + f, \tag{S6}$$

$$\partial_t a = D_a r^{-2}\partial_{xx} a + x\phi(r)\partial_x a + g + g_D, \tag{S7}$$

where $x \in [0,1]$ and $\phi(r) = \dot{r}(t)r(t)^{-1}$. Reaction functions f, g are non-linear and depend on tissue maturation, see Section S1.2.2. Function g_D describes distal-field up-regulation of the activator and is limited to an immature region near the growing end of the domain, see Section S1.2.3. Diffusivities D_i, D_a are such that $D_i \gg D_a$, which is a usual condition of Turing instability – long range inhibitor and short range activator. Without loss of generality, variables a, i describe deviations from a constant equilibrium state, which we standardize to $i^o = a^o = 0$.

We introduce tissue maturation m to make the transition between the Turing and the bi-stable regimes. It is a continuous, space-time dependent variable evolving according to:

$$\partial_t m = x\phi(r)\partial_x m + (\alpha_n + \alpha_a \mathbb{I}_{[a(x_\tau,\tau)>a_{m^*}]})m(1-m). \tag{S8}$$

Fully matured tissue is at $x = 0$ and the less immature at $x = r$. Increase of maturation is due to two effects: intrinsic with a constant rate α_n and promoted by the activator with rate α_a when its concentration reaches a threshold a_{m^*}. Time delay τ assures that signaling centers form in the anterior to posterior direction. Otherwise, distal part of the tissue would mature immediately after the new burst of the activator and a traveling wave could not propagate. Due to the domain scaling, the activator concentration is computed at x_τ, which satisfies $x_\tau r(t-\tau) = xr(t)$.

S1.2.2 Reaction kinetics

To define reaction kinetics of the activator and the inhibitor, we follow the minimal, piecewise linear toy model studied by Crampin et al. in [5, 4],

$$f(i,a,m) = -\sigma(m)i + a, \tag{S9}$$

$$g(i,a,m) = \begin{cases} g_1 \\ g_2 \\ g_3 \end{cases} = \begin{cases} -i - \mu(a + 2Q_l), & a < -Q_l \\ -i + \mu a, & -Q_l \leq a \leq Q_h \\ -i - \mu(a - 2Q_h), & Q_h < a \end{cases}. \tag{S10}$$

Inclinations of the $f, g-$ nullclines, presented in Figure S1, are characterized by positive parameters σ and μ respectively, which model rates of auto-inhibition of the inhibitor and auto-catalysis of the activator. Threshold activator concentrations $Q_l, Q_h > 0$ correspond to saturation and depletion levels and separate the three branches of the g function.

The relation between parameters σ and μ determines the behavior of the activator-inhibitor system:

- Turing regime: If $\mu\sigma < 1$, then there is only one stable equilibrium state, here $E^o = (a^o, i^o) = (0,0)$, that in the presence of diffusion can evolve into a spatial pattern if system parameters satisfy Turing instability conditions.

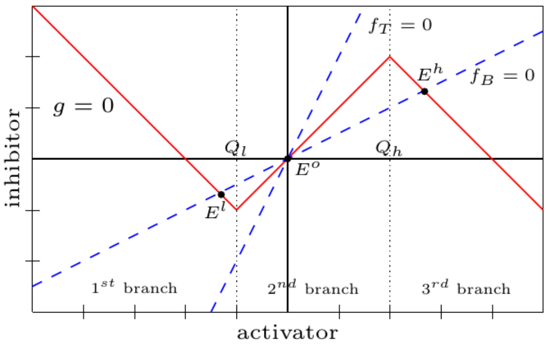

Figure S1: Nullclines of the activator-inhibitor reaction functions $f = g = 0$. By changing the slope σ we regulate if the nullclines cross only in the Turing regime ($f_T = g$ at E^0) or additionally in the bi-stable regime ($f_B = g$ at E^0, E^l, E^h). Concentrations Q_l, Q_h denote activator depletion and saturation thresholds respectively.

- Bi-stable regime: If $\mu\sigma > 1$, then there are two additional stable states, here $E^{l,h} = (a^{l,h}, i^{l,h})$, on the first and the third branch respectively. A traveling wave can evolve from a spatially heterogeneous configuration. The speed of the wave depends on the difference between the stability of these states. The up-regulation wave from distal to mesial regions is observed if the high regulated state E^h is *more stable* than the low regulated state E^l, that is when $Q_l < Q_h$.

Transition between these two regimes depends on the level of maturation of the tissue up to one parameter. To make it simple, we assume that the parameter σ can take two values depending on the maturation m:

$$\sigma = \sigma(m) = \begin{cases} \sigma_{\mathrm{T}} & \text{if } m > m^*_\sigma \in [0,1] \\ \sigma_{\mathrm{B}} & \text{otherwise} \end{cases}, \tag{S11}$$

where $\mu\sigma_B > 1$ is such that low maturation is associated with bi-stability (and moving interfaces), and $\mu\sigma_T < 1$ with suitable conditions on the parameters are such that high maturation is associated with Turing instability.

S1.2.3 Distal field activation

Formation of each tooth is preceded by a burst of activator in the distal region of dental epithelium, but the mechanism of these successive up-regulations remains unknown. In our modeling approach, we propose extrinsic oscillations, which are simply modeled by a two-components system with self-sustained oscillations. We introduce a mesenchyme signaling molecules s that interact with the activator a: it is produced by the activator with rate $\gamma > 0$ on sufficiently immature tissue ($m < m^*_d$)

$$\partial_t s = x\phi(r)\partial_x s + \gamma a \cdot \mathbb{I}_{m<m^*_d}, \tag{S12}$$

and it has a negative feedback on the activator concentration

$$g_D = \begin{cases} k_s & s < s_L, m < m_d^* \\ -k_s & s > s_H, m < m_d^* \\ 0 & \text{otherwise} \end{cases}, \quad \text{(S13)}$$

with $k_s > 0$ and $s_L < s_H$.

The oscillating mechanism works as follows: up-regulated activator a increases the concentration of s, which after reaching the high threshold value s_H, down-regulates a (switch off); then production of s falls and its concentration decreases up to the low threshold s_L, which allows to up-regulate a again (switch on).

S1.3 Modeling teeth fusion

In wild-type development the R2 signaling center does not develop an individual tooth, because after recovering from the developmental palimpsest it joins M1 to form one, large tooth. Research on hair development shows that Edar pathway plays a role in cell migration, so it may influence the movement of R2 and M1 towards each other. But how Edar expression, initially localized in two separate spots, is spread in the area between R2 and M1 is unknown. In what follows, we describe modeling approach to test the hypothesis that the R2-M1 fusion phenomena can result from cell migration mediated by chemotaxis interacting with the Turing pattern.

S1.3.1 Chemotaxis model

We consider a fixed, fully matured domain $\Omega = [0, L]$, $m(t, x) = 1$ and activator-inhibitor reactions in the Turing regime with parameters such that a two-spots pattern is admissible. We assume that the chemotaxis mechanism is based on the interaction between motile cells and a chemical substance that attracts them, [8, 10].

Expression of Edar in dental epithelium coincides with high concentrations of activator, so it is straightforward to consider it as a chemoattractant for cells, but how cells affect activator-inhibitor concentrations is unclear. In order to observe the fusion of R2 and M1, activator up-regulation between two, stable peaks should be promoted. This positive feedback may be direct, or indirect through a negative feedback on the inhibitor. We tested both scenarios, but the former never resulted in the fusion of signaling centers, whereas the latter can lead to the fusion, but not always. Our interpretation is that activator is a short-range diffusive molecule, so it can not mediate a positive attraction between the two peaks, on the contrary to inhibitor. In our model, in order to increase activator concentration, cells reduce inhibitor level.

We describe cells at the continuous level through macroscopic density c. We assume that they diffuse with diffusivity $D_c > 0$ and respond to gradients in activator concentration with sensitivity $\chi > 0$ moving towards sources of activator. In regions where cells density reaches a threshold c^*, inhibitor concentration is decreased with rate $\alpha > 0$. Under these hypotheses, the activator-inhibitor-cells

chemotaxis system writes as

$$\partial_t i = D_i \partial_{xx} i + f - \alpha c \cdot \mathbb{I}_{[c>c^*]}, \tag{S14}$$

$$\partial_t a = D_a \partial_{xx} a + g, \tag{S15}$$

$$\partial_t c = D_c \partial_{xx} c - \chi \partial_x \left(c \left(1 - \frac{c}{c_{\max}} \right) \partial_x a \right), \tag{S16}$$

where reaction terms f, g are given by (S9)-(S10). The logistic saturation in the chemoattractant gradient response prevents formation of high cell density area, as the motility is blocked above a certain threshold $c_{\max}$, see [7].

S1.4 Initial and boundary conditions

We complete the model of signaling centers patterning, Section S1.2, with the homogeneous Neumann and Dirichlet boundary conditions as follows

$$\begin{array}{ll} \partial_x i = 0, \quad a = -1, & \text{at } x = 0, t \geq 0, \\ \partial_x i = \partial_x a = \partial_x s = m = 0, & \text{at } x = r(t), t \geq 0, \end{array} \tag{S17}$$

and the initial conditions

$$i_0 = 0, \tag{S18}$$

$$a_0 = -1 + 2\frac{\arctan(25x)}{\arctan(25)}, \tag{S19}$$

$$s_0 = s_L + 0.5(s_H - s_L), \tag{S20}$$

$$m_0 = \frac{0.5\pi + \arctan(-15x + 5)}{0.5\pi + \arctan(5)}. \tag{S21}$$

Dirichlet boundary condition for the activator prevents formation of a pattern with a maximum point at the boundary (S17).

We begin with a mostly immature region at initial time (S21). This corresponds to the bi-stable regime. We prescribe initially an activator which is up-regulated everywhere but in a small boundary layer close to the left boundary at $x = 0$ (S19), consistently with the Dirichlet boundary condition imposed there at any positive time (S17).

In case of the chemotaxis model, Section S1.3, we set homogeneous Neumann boundary conditions for all components and

$$i_0 = 0, a_0 = 1 + 0.1 \sin(2\pi x/L)^2, c_0 = \frac{M_c}{L},$$

where M_c is the total mass of cells.

S2 Simulations

The mathematical model that we propose can reproduce formation of stable patterns (in Turing regime), traveling waves (in bi-stable regime), distal bursts of activator and developmental palimpsest. We note that model calibration is empirical and that the aim of numerical simulations is to qualitatively reproduce experimental behavior. The main difficulty is that sequential patterning of teeth consists in a succession of elementary patterns and requires accurate temporal synchronization of their dynamics, such as:

- The size of the initial mature region is determinant for the patterning . If it is too small, that is smaller than the Turing pattern wave length, then the first spot can not be confined and stabilized and we observe a traveling wave propagating from mature (Turing) to immature (bi-stable) region. If it is too large, then Turing conditions on the mature tissue prevent from developmental palimpsest, because traveling wave can not reach and destroy the pattern.
- Shorter wavelength of the Turing pattern allows to use smaller initial mature region, but then the pattern is stronger and more difficult to be perturbed by the wave.
- Larger stability difference between the depletion Q_l and the saturation Q_h states increases the wave speed, but it also increases Turing wavelength. The latter requires larger mature tissue for the first pattern and, as previously, may prevent from the developmental palimpsest.
- Distal activation/deactivation has to be synchronized with the tissue growth. Too early down-regulation (small s_H) results in insufficient activator enhanced maturation and a pattern can not be stabilized. Too late upregulation starts traveling wave too far, which gives time for tissue maturation and prevents from the developmental palimpsest.

In order to perform numerical simulations we discretized the maturation and the mesenchyme signal advection-reaction equations (S8),(S12) with a classical explicit upwind method. Activator, inhibitor and cells density are given by advection-diffusion-reaction equations and we applied the Scharfetter-Gummel type finite volume method in space [11] and implicit-explicit (IMEX) in time. All details of the numerical approximation are presented in [2], where we performed numerical convergence analysis and showed that these schemes are sufficient to accurately simulate our mathematical model.

S2.1 Numerical results

We explored parameter space in the aim to prove that several scenarios of teeth patterning can exist. In Table 1 we present values used in the simulations with the following notation: F_1 - sequential patterning without traveling wave (Figure 2), F_2 - wild-type development (Figure 3), F_3 - mutant development (Figure 4) and $M_{1,2,3}$ - chemotaxis test for respectively small domain-small chemotaxis, small domain-strong chemotaxis and large domain-strong chemotaxis (Figure 6).

Parameter	Definition	Value:$(\mathbf{F_1}, \mathbf{F_2}, \mathbf{F_3}, \mathbf{M_1}, \mathbf{M_2}, \mathbf{M_3})$
L	Domain size	$r(t), r(t), r(t), 2.6, 2.6, 3$
D_i	Diffusivity of inhibitor	(1)
D_a	Diffusivity of activator	(0.01)
D_c	Diffusivity of cells	$-,-,-,(0.02)$
σ_T	Rate of auto-inhibition of inhibitor (Turing)	$0.55, 0.55, 0.39, (0.55)$
σ_B	Rate of auto-inhibition of inhibitor (bi-stable)	$20, 20, 6, -,-,-,$
μ	Rate of auto-activation of activator	$0.4, 0.4, 0.4, (0.55)$
m_σ^*	Maturation threshold for Turing/bi-stable regime	(0.7)
Q_l	Depletion level of activator	(0.7)
Q_h	Saturation level of activator	$0.7, 1, 1, (0.7)$
λ_G	Rate of domain growth	(0.005)
α_n	Rate of spontaneous maturation	$(0.0005), -,-,-$
α_a	Rate of activator enhanced maturation	$(0.1), -,-,-$
a_m^*	Activator threshold for enhanced maturation	$(0.9), -,-,-$
τ	Delay time of enhanced maturation	$(100), -,-,-$
k_s	Rate of activator production by mesenchyme signal	$(10), -,-,-$
s_L	Low mesenchyme signal threshold	$(1.1), -,-,-$
s_H	High mesenchyme signal threshold	$(1.4), -,-,-$
m_d^*	Maturation threshold for distal activation	$(0.1), -,-,-$
γ	Rate of production of mesenchyme signal	$(0.001), -,-,-$
χ	Chemo-sensitivity of cells	$-,-,-, 0.1, 0.5, 0.5$
$c_{\max}$	Maximal cells density	$-,-,-,(3)$
c^*	Cells density threshold for inhibitor degradation	$-,-,-,(2.9)$
α	Rate of inhibitor degradation by cells	$-,-,-,(0.3)$

Table 1: Parameters used in numerical simulations: F_1 - sequential patterning without traveling wave, F_2 - wild-type development, F_3 - mutant development and $M_{1,2,3}$ - fusion for respectively small domain – small chemotaxis, small domain – strong chemotaxis and large domain – strong chemotaxis. When possible, we denote by $(\cdot)$ values that are the same for more than one simulation.

In particular, we were able to determine a range of parameters for which the pattern develop exactly in the correct order. Moreover, through the parameter sensitivity analysis we show that our model is robust for the chosen set of parameters. Wild-type development is recovered even if the values are significantly perturbed. Figures S2-S4 present the effect of the variations of several parameter values (one at a time) by $\pm 10\%, \pm 50\%$ from the those in Table 1. Additionally, variations in some of them can disrupt the developmental wild-type program, and can correspond to some mutants. For example, decrease of the auto-inhibition of the inhibitor by 25% leads to the increase of the inhibitor concentration, which prevents from developmental palimpsests and preserves the R2 signaling center. For other possible mutant scenarios see [2].

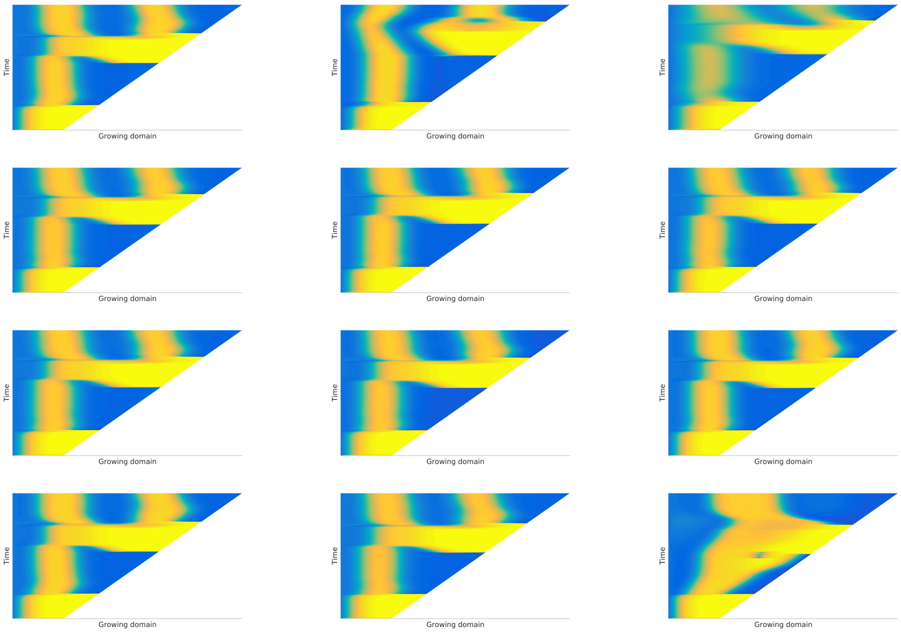

(a) σ_T (b) σ_B (c) μ

Figure S2: Sensitivity analysis of activator-inhibitor reaction rates parameters. Figures present time evolution of the activator concentration for variations of parameters values by $-50\%, -10\%, 10\%, 50\%$ (from top to bottom) from values in Table 1: (a) σ_T - rate of auto-inhibition of the inhibitor i in the Turing regime, (b) σ_B - rate of auto-inhibition of the inhibitor i in the bistable regime, (c) μ - rate of auto-catalysis of the activator a.

References

[1] N. H. Barton and M. Turelli. Spatial waves of advance with bistable dynamics: Cytoplasmic and genetic analogues of allee effects. *The American Naturalist*, 178(3):E48–E75, 2011.

[2] V. Calvez, S. Pantalacci, and M. Twarogowska. Mathematical modeling of early teeth pattering. in prep.

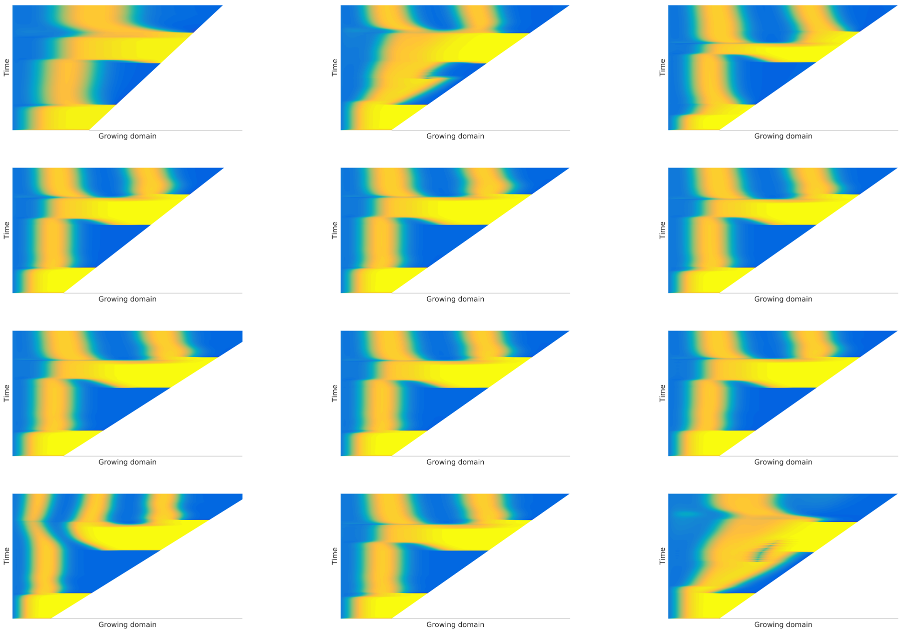

(a) λ_G (b) α_a (c) τ

Figure S3: Sensitivity analysis of tissue parameters. Figures present time evolution of the activator concentration for variations of parameters values by $-50\%, -10\%, 10\%, 50\%$ (from top to bottom) from values in Table 1: (a) λ_G - rate of domain growth, (b) α_a - rate of activator enhanced maturation, (c) τ - delay time.

[3] E. J. Crampin, E. A. Gaffney, and P. K. Maini. Reaction and diffusion on growing domains: Scenarios for robust pattern formation. *Bulletin of Mathematical Biology*, 61(6):1093 – 1120, 1999.

[4] E. J. Crampin, E. A. Gaffney, and P. K. Maini. Mode-doubling and tripling in reaction-diffusion patterns on growing domains: A piecewise linear model. *Journal of Mathematical Biology*, 44(2):107–128, 2002.

[5] E. J. Crampin, W. W. Hackborn, and P. K. Maini. Pattern formation in reactiondiffusion models with nonuniform domain growth. *Bulletin of Mathematical Biology*, 64(4):747 – 769, 2002.

[6] A. Gierer and H. Meinhardt. A theory of biological pattern formation. *Kybernetik*, 12:30–39, 1972.

[7] T. Hillen and K. J. Painter. A user's guide to pde models for chemotaxis. *Journal of Mathematical Biology*, 58(1), 2008.

[8] E. F. Keller and L. A. Segel. Initiation of slime mold aggregation viewed as an instability. *Journal of Theoretical Biology*, 26(3):399 – 415, 1970.

[9] P. M. Kulesa, G. C. Cruywagen, S. R. Lubkin, P. K. Main, J. Sneyd, M. W. J. Ferguson, and J. D. Murray. On a model mechanism for the

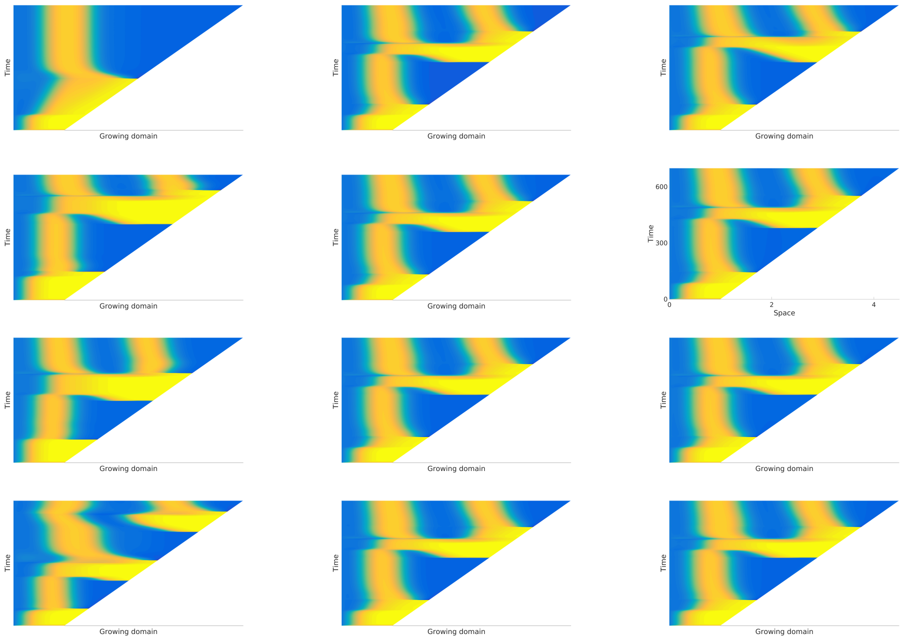

(a) γ (b) k_s (c) m_ε^*

Figure S4: Sensitivity analysis of mesenchyme parameters. Figures present time evolution of the activator concentration for variations of parameters values by $-50\%, -10\%, 10\%, 50\%$ (from top to bottom) from values in Table 1: (a) γ - rate of production of mesenchyme signal (b) k_s - rate of degradation of activator by mesenchyme signal, (c) m_ε^* - maturation threshold for distal activation.

spatial patterning of teeth primordia in the alligator. *Journal of Theoretical Biology*, 180(4):287 – 296, 1996.

[10] C. Patlak. Random walk with persistence and external bias. *The bulletin of mathematical biophysics*, 15(3):311338, 1953.

[11] D. L. Scharfetter and H. K. Gummel. Large-signal analysis of a silicon read diode oscillator. *IEEE Transactions on Electron Devices*, 16(1):64–77, 1969.

[12] A. Turing. The chemical basis of morphogenesis. *Philosophical Transactions of the Royal Society B*, 237:37–72, 1952.
