# Supplementary figures and images for: Modeling Edar expression reveals the hidden dynamics of tooth signaling center patterning
Source: PLoS Biol. 2019 Feb 7;17(2):e3000064. doi: 10.1371/journal.pbio.3000064 (PMC6382175; doi:10.1371/journal.pbio.3000064)

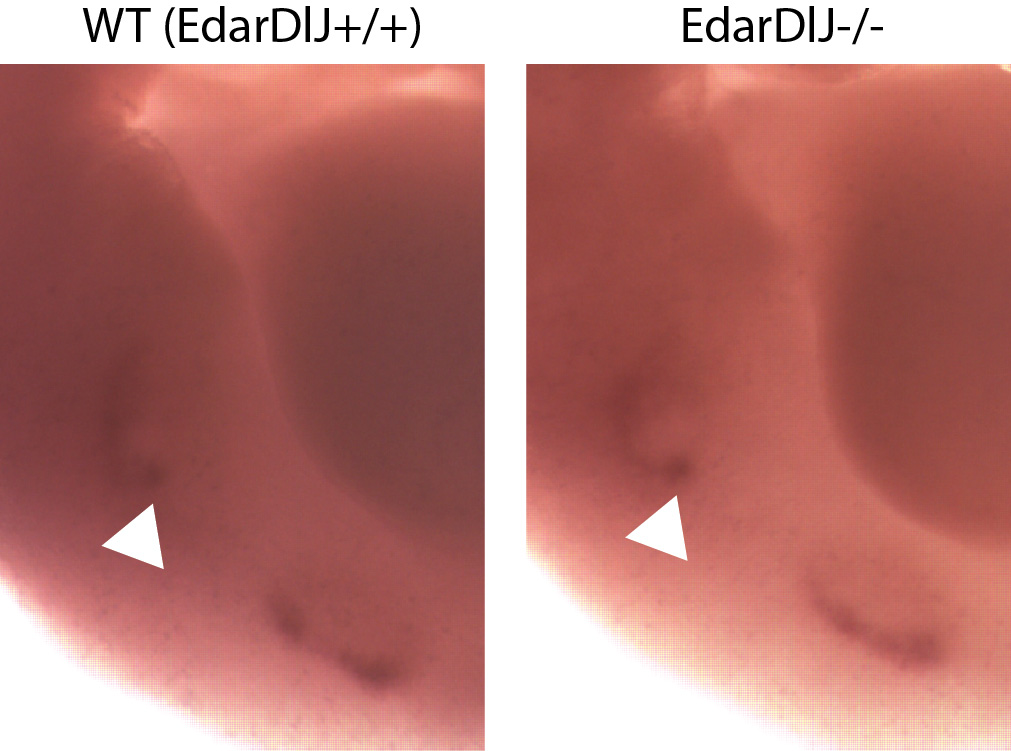

Supplement: S1 Fig — dpc, days post coitum; EdardlJ, EdardownlessJ. (JPG) [file pbio.3000064.s003.jpg]

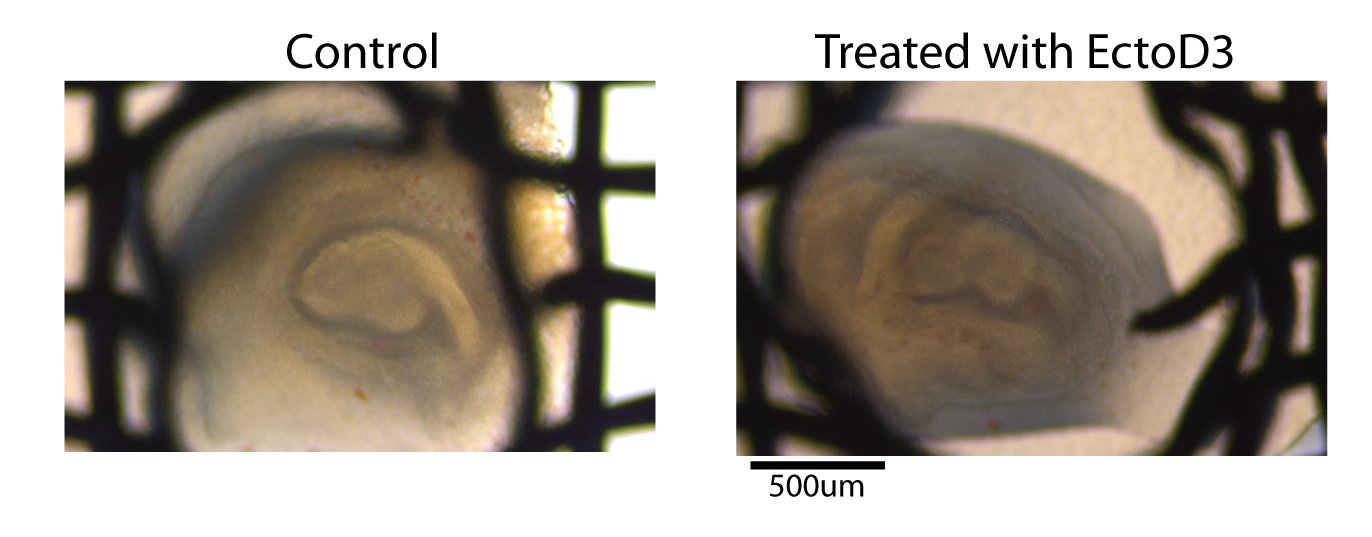

Supplement: S2 Fig — (TIF) [file pbio.3000064.s004.tif]

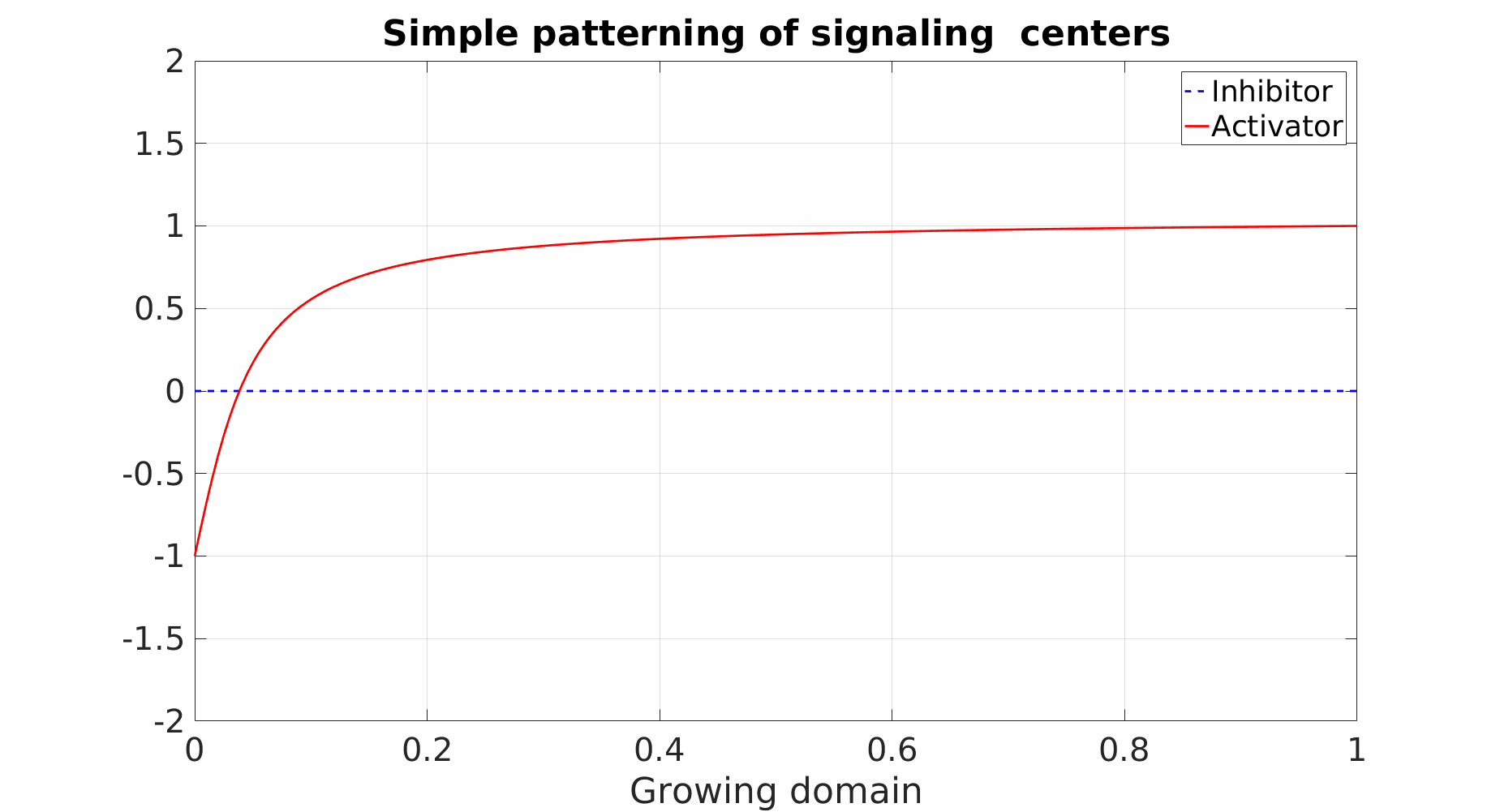

Supplement: S1 Movie — The evolution of activator concentration (red), inhibitor concentration (blue), and domain maturation (green) is shown as the domain grows. (GIF) [file pbio.3000064.s005.gif]

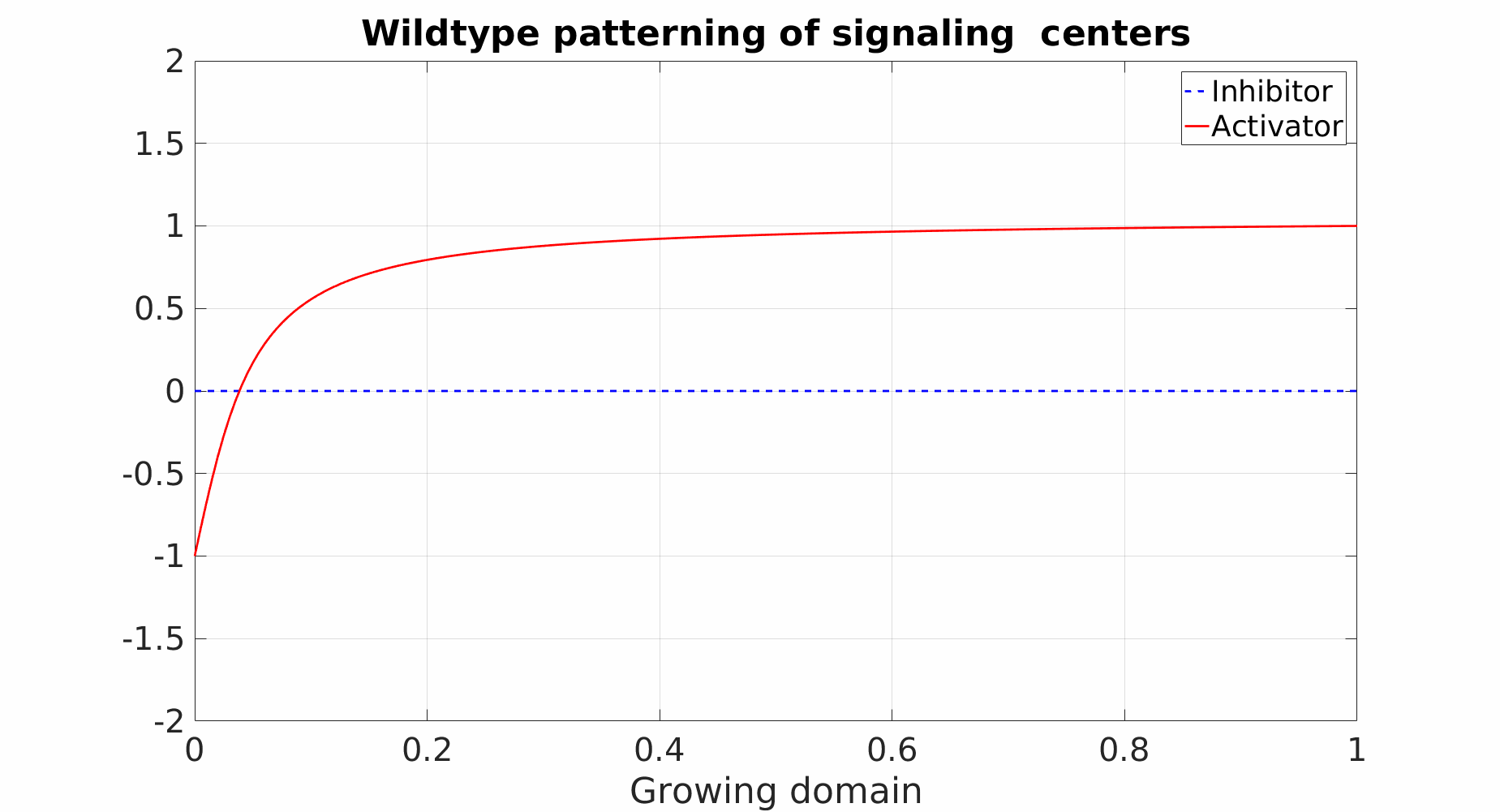

Supplement: S2 Movie — The evolution of activator concentration (red), inhibitor concentration (blue), and domain maturation (green) is shown as the domain grows. (GIF) [file pbio.3000064.s006.gif]

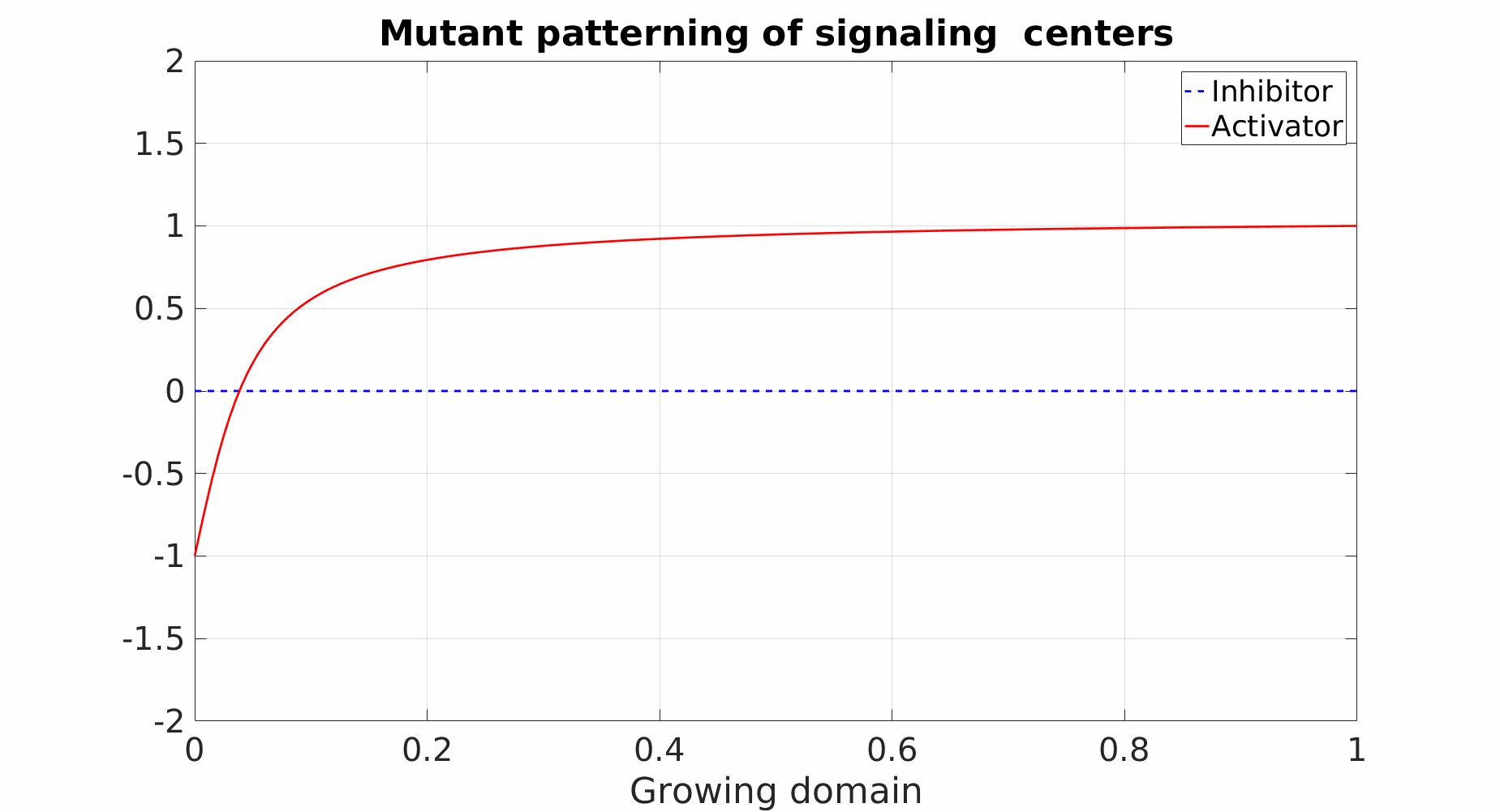

Supplement: S3 Movie — The evolution of activator concentration (red), inhibitor concentration (blue), and domain maturation (green) is shown as the domain grows. (GIF) [file pbio.3000064.s007.gif]
